# Chemical Components and Hypouricemic Activity Monitoring of *Astragali radix*-Huaier During Fermentation Processing Using High-Resolution Mass Spectrometry Combined with Untargeted Metabolomics

**DOI:** 10.3390/foods15101758

**Published:** 2026-05-15

**Authors:** Zhicheng Yin, Jie Li, Shuyi Song, Hong Wang, Tianmei Niu, Xiaojie Wang, Shengqian Sun, Jiayu Zhang

**Affiliations:** 1 School of Traditional Chinese Medicine, Shandong Medical and Pharmaceutical University, Yantai 264003, China; 15650083775@163.com (Z.Y.); lijie123xyd@163.com (J.L.); songshuyi1013@163.com (S.S.); wanghong20201002@163.com (H.W.); niutianmei123@163.com (T.N.); 2 College of Pharmaceutical Science, Shandong University of Traditional Chinese Medicine, Jinan 250355, China; 3 College of Life Sciences, Shandong Agricultural University, Taian 271018, China; 4 Yantai Key Laboratory of Special Medical Food, Yantai Institute of Technology, Yantai 264003, China; sunshengqian@yitsd.edu.cn; 5 Research & Development Center, Jiangsu Matchwell Pet Products Supply Co., Ltd., Suqian 223800, China

**Keywords:** *Astragali radix*-Huaier fermentation, bidirectional solid fermentation, metabolite identification, metabolomics, lowering uric acid

## Abstract

Recent evidence highlights the therapeutic potential of *Astragali radix*-Huaier fermentation products for hyperuricemia treatment, although the dynamics of the fermentation process remain poorly understood. This study employed high-resolution mass spectrometry and untargeted metabolomics for real-time monitoring of chemical components and hypouricemic activity throughout fermentation. The results revealed significant alterations in the chemical composition, with distinct sample separations observed on days 7, 14, 21, and 28. A total of 33 differential components were identified, including 20 flavonoids and 13 saponins, eight of which showed notable changes. Polysaccharides and saponins were found to correlate positively with the uric acid-lowering effect. On day 21, the levels of total polysaccharides and cycloastragenol-6-glucoside, a saponin derivative, peaked, coinciding with the highest hypouricemic activity of the *Astragalus* fungal fermentation products. This study provides the first evidence of dynamic changes in the chemical profile and pharmacological activity of *Astragali radix*-Huaier during fermentation, paving the way for optimizing fermentation processes and developing medicinal and dietary products based on *Astragali radix*.

## 1. Introduction

*Astragali radix* (AR), the dried root of *Astragalus membranaceus* (Fisch.) Bge. var. *mongholicus* (Bge.) Hsiao or *Astragalus membranaceus* (Fisch.) Bge. [1,2,3], has historically been used as both a spice and a food additive across East Asia and the Middle East for thousands of years [4]. Recently, it was included in the list of edible herbs by the Chinese Ministry of Health and legally recognized as a dietary supplement under the U.S. Dietary Supplement Health and Education Act [5,6]. This growing recognition highlights AR’s dual role in traditional Chinese medicine (TCM) as both a medicinal and culinary herb. This dual-use paradigm is formally recognized as “food and medicine homology” (FMH) [7], a concept increasingly endorsed by contemporary scientific frameworks [8]. In 2026, the Chinese National Health Commission and the State Administration for Market Regulation formally incorporated AR into the official FMH directory, thereby affirming its regulatory status as a substance that may be used both as a food ingredient and as a medicinal material. This designation marks a pivotal shift: AR is no longer confined to the domain of traditional Chinese medicine (TCM) but is emerging as a functional ingredient with validated applications in modern food systems [9]. Among emerging processing strategies, fermentation technology offers a particularly promising solution for FMH substances [10].

During fermentation, microbial enzymes, including glycosidases, esterases, and decarboxylases, catalyze the biotransformation of complex bioactive compounds into smaller, more absorbable metabolites with enhanced solubility, stability, and gastrointestinal absorption [11]. This enzymatic degradation reduces molecular weight, modifies chemical structures, and often generates novel bioactive metabolites with improved pharmacological profiles. Furthermore, fermentation with Huaier significantly enhances the transformation of chemical components, resulting in notable changes in biological activity [12,13]. For AR specifically, bidirectional solid-state fermentation with medicinal fungi such as Huaier has been shown to degrade cellulosic matrices, release bound bioactive compounds, and convert glycosylated precursors into more bioavailable aglycone forms. These transformations directly address the bioavailability limitations that have historically constrained the use of AR in food applications [14].

Recent research on reducing UA in asymptomatic hyperuricemia has increasingly focused on innovative bidirectional solid fermentation techniques [15,16,17]. For example, a study demonstrated that *Bacillus subtilis*-fermented AR alleviates hyperuricemia by modulating the gut microbiota [5,18]. This fermentation strategy not only produces novel pharmacological effects but also shows promise in mitigating UA accumulation and diabetic nephropathy through microbiota regulation. By prioritizing renal protection, this approach offers the dual benefit of lowering UA levels while maintaining renal homeostasis [19,20]. Our preliminary findings further confirmed the significant UA-lowering effects of fermented AR and elucidated its mechanism in treating hyperuricemia through the integration of metabolomics and transcriptomics [21]. Fermentation technology, widely employed in food and health products, enhances bioavailability and improves taste. The bioactive components of AR, such as polysaccharides, saponins, and flavonoids, are recognized for their renal protective properties [22,23]. Fermentation specifically addresses the absorption limitations of macromolecules [21]. In this study, the fermentation of AR with Huaier harnesses Huaier’s degradation capacity, thereby enhancing the traditional applications of AR and contributing to the richness of TCM culture.

The limited bioavailability of Huaier fruiting bodies restricts their commercial and industrial potential [24]. However, in a bidirectional solid fermentation process [25], medicinal fungi interact with microorganisms capable of degrading cellulose and hemicellulose in AR. This interaction significantly enhances product quality by inducing metabolic changes in flavonoids and saponins, thereby broadening their potential applications. Traditional fermentation optimization methods, however, lack real-time monitoring of pharmacological effects. Addressing quality control (QC) challenges in the fermentation of AR and Huaier is crucial for improving bioconversion efficiency and maximizing the utilization of active ingredients. High-resolution mass spectrometry (HRMS) technology [26] provides valuable support for process optimization, offering non-destructive capture of spectral and image data. Additionally, non-targeted metabolomics allows for the detection of unknown metabolites in complex biological samples during fermentation, enabling real-time monitoring.

This study applied HRMS technology and non-targeted metabolomics to investigate the impact of compositional changes during the bidirectional solid fermentation of AR and Huaier, with a focus on their UA-lowering properties. Ultra-high-performance liquid chromatography–tandem mass spectrometry (UHPLC-Q-Exactive Orbitrap MS) was coupled with metabolomics to track compositional changes throughout fermentation. High-performance liquid chromatography (HPLC) was used to monitor specific component changes in fungal substances (FS0, FS7, FS14, FS21, and FS28) at different fermentation stages, and their conversion effects were validated through separate fermentation experiments. The UA-lowering effects of components isolated at various fermentation stages were assessed using the HK-2 hyperuricemia cell model [27]. Pharmacodynamic indicators were employed to track compositional changes during the optimal fermentation process [28]. Previous studies investigating the fermentation of AR have reported enhancements in various activities, such as immunomodulation and nephroprotection. However, these studies primarily focused on the fermentation process and pharmacological research, while neglecting the correlation between changes in chemical composition and biological activity. Furthermore, there has been no research on the fermentation of AR using Huaier. Therefore, this study provides a comprehensive evaluation of the fermentation process of AR and Huaier, focusing on compositional and efficacy changes to elucidate the conversion mechanisms of key components in AR. The findings provide a solid foundation for systematically exploring its UA-lowering mechanism and offer valuable insights for advancing microbial fermentation engineering, paving the way for further development of AR research.

## 2. Materials and Methods

### 2.1. Chemicals and Reagents

AR was sourced from Shanxi Hunyuan Wansheng Astragalus Development Co., Ltd. (Hunyuan County, Datong, China) (NO. SX20191101), and Huaier was sourced from Beijing Biowei Biotechnology Co., Ltd. (Beijing, China) (NO. Bio-20449). Calycosin-7-glucoside, calycosin, ononin, formononetin, astragaloside I, astragaloside II, astragaloside IV, and D-glucose were purchased from Must Bio-tech Co., Ltd. (Chengdu, China). Cycloastragenol-6-glucoside was obtained from Chengdu Biopurify Phytochemicals Ltd. (Chengdu, China). All reference standards were analyzed by HPLC and their purities exceeded 98%. Methanol, acetonitrile, ethanol, and formic acid of HPLC grade were acquired from Tianjin Kemiou Chemical Reagent Co., Ltd. (Tianjin, China). Purified water was obtained from Wahaha Group Co., Ltd. (Hangzhou, China). The human renal proximal tubular epithelial cell line (HK-2) was purchased from Wuhan Pricella Biotechnology Co., Ltd. (Wuhan, China).

### 2.2. Preparation of Fermentation Products

#### 2.2.1. Fermentation Strategy and Sample Preparation

The optimal fermentation conditions (including temperature, particle size, inoculation ratio, water-to-material ratio, and bagging time) were determined through process optimization (Supplementary Materials S1). 10 mL of distilled water was added to a fermentation bag containing 20 g of AR powder, which was sterilized under high-pressure steam at 121 °C for 15 min. After cooling, 30 mL of Huaier seed liquid was inoculated into the mixture, and cultivation was conducted at 28 °C in an incubator. The fermentation products were collected on the 7th, 14th, 21st, and 28th days, dried, pulverized, and sieved for use (*n* > 6 per group). The products from each fermentation stage were labeled as FS0, FS7, FS14, FS21, and FS28, respectively.

#### 2.2.2. Preparation of Test Samples

For analysis, 1.0 g of powder from each sample (FS0, FS7, FS14, FS21, and FS28; *n* = 3 per group) was dissolved in 50 mL of methanol and heated under reflux for 2 h. After filtration, 25.0 mL of the solution was concentrated. The residue was then transferred to a 5.0 mL volumetric flask and diluted to the mark with methanol. QC samples were prepared using the relevant method. Flavonoid standards (including calycosin-7-glucoside, calycosin, ononin, and formononetin) and saponins (such as astragaloside I, astragaloside II, astragaloside IV, and cycloastragenol-6-glucoside) were prepared as 1 mg/mL solutions. Both the extract and standard solutions were filtered through a 0.45 μm microporous membrane filter before analysis. All sample solutions were stored at 4 °C.

All experiments were performed with independent biological replicates as follows: for fermentation sample preparation, *n* = 6 biologically independent fermentation batches per time point (FS0, FS7, FS14, FS21, FS28); for chemical analysis (metabolomics and HPLC quantification), each biological replicate was analyzed with *n* = 3 technical replicates; for cell-based efficacy assays (HK-2 cell model), *n* = 4 biological replicates (independent cell passages) were used, each with triplicate technical wells. Data are presented as the mean ± SD of biological replicates unless otherwise specified.

### 2.3. Metabolomics Information Collection and Analysis

Metabolomic analysis was performed on all samples, including the non-fermented control (FS0) and fermented products (FS7, FS14, FS21, FS28). UHPLC analysis was performed using a DIONEX Ultimate 3000 UHPLC system (Thermo Fisher Scientific, Waltham, MA, USA), equipped with a binary pump, automated sampler, and column compartment. Chromatographic separation was carried out on a Waters ACQUITY BEH C_18_ column (2.1 × 100 mm, 1.7 μm). The mobile phase consisted of 0.1% formic acid aqueous solution (A) and acetonitrile (B), with a flow rate of 0.3 mL/min. The linear gradient procedure was as follows: 0–8 min, 5→20% (B); 8–10 min, 20→30% (B); 10–12 min, 30→35% (B); 12–14 min, 35→40% (B); 14–23 min, 40→55% (B); 23–27 min, 55→95% (B); 27–28 min, 95→5% (B); 28–30 min, 5% (B). The injection volume was 5.0 μL.

The HRMS and MS/MS spectra were acquired using a Q-Exactive Orbitrap mass spectrometer with electrospray ionization (ESI) (Thermo Fisher Scientific, Waltham, MA, USA). The operating parameters were as follows: nitrogen (purity ≥ 99.99%) was used as both the sheath gas and auxiliary gas. The sheath gas flow was set at 35 arb, while the auxiliary gas flow was set to 10 arb. The capillary temperature was maintained at 320 °C, the probe heater temperature at 350 °C, and the cone voltage for ESI was set to 3800/3500 V (+/−). High-resolution mass spectra were obtained by the Orbitrap analyzer using a full scan in the *m*/*z* 80–1200 range, with a resolution of 70,000 in MS mode and 17,500 in dd MS/MS mode.

Principal component analysis (PCA) was performed on the data using Simca software, version 14.1 (Umetrics AB, Umea, Sweden). Hierarchical Cluster Analysis (HCA) was subsequently applied based on PCA results. Supervised orthogonal partial least squares discriminant analysis (OPLS-DA) was used to assess the Variable Importance in Projection (VIP) and S-plots, identifying key marker compounds contributing to classification. Differential metabolites were identified by integrating the results with compound standards and literature data. Flavonoids and saponins were quantified through standard comparison. The relative peak areas of identified metabolites were summarized and compared across groups, with components showing significant content changes (*p* < 0.0001) identified.

### 2.4. Analysis of Differential Metabolite Composition Monitoring

#### 2.4.1. Quantitative Analysis of Flavonoids, Saponins, and Polysaccharides by HPLC

HPLC-ELSD and HPLC-UV analyses of fermented AR-Huaier samples were performed using a Shimadzu LC-20AT high-performance liquid chromatograph (Shimadzu Corporation, Kyoto, Japan) equipped with an SPD-20A UV detector, an ELSD, and an autosampler. Flavonoids were separated on a Kromasil 100-5-C18 column (250 mm × 4.6 mm, 5 μm) (Kromasil, Bohus, Sweden) with a mobile phase consisting of 0.2% formic acid aqueous solution (A) and acetonitrile (B). The gradient elution program was as follows: 0–20 min, 20–40% (B); 20–30 min, 40% (B); 30–30.1 min, 40–20% (B); 30.1–40 min, 20% (B). The analysis was performed at a flow rate of 1 mL/min, with a column temperature of 35 °C and a detection wavelength of 260 nm. Additional method details are provided in Supplementary Material S2.

For saponin analysis, HPLC-ELSD was conducted following the method outlined in the Chinese Pharmacopoeia (2025 Edition). The gradient elution program was as follows: 0–10 min, 15%→40% (B); 10–20 min, 40%→60% (B); 20–30 min, 60%→85% (B); 30–30.1 min, 85%→15% (B); 30.1–40 min, 15% (B). The ELSD parameters were: flow rate 1 mL/min, nebulizer tube temperature 55 °C, and gas flow rate 2.5 mL/min. Data collection and analysis were performed using the LabSolution workstation (Shimadzu Corporation).

The polysaccharide component [29] with the highest concentration during fermentation of AR-Huaier was isolated from samples labeled FS0, FS7, FS14, FS21, and FS28. Each sample, weighing 5 g, was mixed with 15 volumes of distilled water and subjected to reflux heating twice for 1.5 h each. The extracts were then pooled, concentrated, alcohol-precipitated, and freeze-dried to obtain crude polysaccharides from the fermented AR mycelium at various fermentation stages. These crude polysaccharides were reconstituted in 10 mL of distilled water, and temporal variations in total sugar content during fermentation were monitored using a standard method (Supplementary Materials S3) for total sugar determination [29]. In this study, the PMP pre-column derivatization method was employed to analyze variations in monosaccharide composition during fermentation. Additionally, changes in the distribution of polysaccharides were assessed using HPLC-ELSD [30,31].

The conditions for monosaccharide composition analysis were as follows: the chromatographic column used was Kromasil 100-5-C18 (250 mm × 4.6 mm, 5 μm). The mobile phase consisted of two components: A) acetonitrile and B) a potassium dihydrogen phosphate buffer (pH 6.7, 50 mmol/L). The elution gradient was set as follows: from 0 to 15 min, 83% B; from 15 to 25 min, a gradient from 83% to 73% B; from 25 to 27.5 min, 73% B; from 27.5 to 40 min, a gradient from 73% to 83% B; and finally, from 40 to 50 min, 83% B. The flow rate was maintained at 1.0 mL/min, the column temperature was set to 35 °C, and the detection wavelength was 250 nm. An injection volume of 20 μL was utilized for the analysis.

The conditions for polysaccharide distribution analysis were as follows: the column used was a BIOBASIC SEC 60 (300 mm × 7.8 mm, 5 μm) (Thermo Fisher Scientific). The analysis was conducted using a high-performance liquid chromatography system equipped with a tandem evaporative light-scattering detector. The mobile phase consisted of pure water, and the column was maintained at a temperature of 35 °C. The carrier gas flow rate was set to 2.5 mL·min^−1^, with a drift tube temperature of 55 °C. An injection volume of 20 μL was utilized for the analysis.

#### 2.4.2. Analysis of the Fermentation and Transformation of Flavonoids and Saponins

The flavonoid components in AR were extracted through a multi-step process (Supplementary Material S2) [32,33]. Briefly, 10-mesh AR was refluxed with 80% ethanol (10 times the volume) for 2 h. This extraction was repeated three times, followed by rinsing the residue with 80% ethanol. The residue (1.0 g) was then subjected to reflux extraction with 50 mL of methanol for 2 h. The resulting extract was filtered, concentrated, and transferred to a 5.0 mL volumetric flask, where it was diluted with methanol and filtered through a 0.22 μm Millipore filter. The flavonoid components were identified using this liquid-phase method. The extraction process was repeated until no further flavonoid components were detected in the AR extract. The final AR residue was reserved for subsequent fermentation. In the quantitative experiment, standard components (calycosin-7-glucoside, calycosin, ononin, and formononetin) were added to the samples and a blank group (without added standards) was included for comparison. All groups underwent three parallel fermentations under optimal conditions (*n* = 6 per group).

Fungal samples aged 0, 3, 6, 8, and 10 days were combined from each fermentation batch. A 50 mL portion of methanol was added to extract the samples by reflux for 2 h. After filtration and concentration, 25 mL of the extract was transferred to a 5.0 mL volumetric flask, diluted with methanol, and filtered through a 0.22 μm Millipore filter. Conversion and content were assessed using methods from Section 2.3 and Section 2.4.1, respectively. To analyze saponin component transformation during fermentation, a monitoring strategy was developed based on the described method, incorporating saponins (astragaloside I, II, and IV). Extracts from each fermentation time point were collected for metabolite and quantitative analysis [34].

### 2.5. Pharmacodynamic Monitoring Based on HK-2 High Uric Acid Cell Model-Variation of Fermentation Components

#### 2.5.1. Preparation of Test Samples

Samples of Huaier mycelia (HM), FS0, FS7, FS14, FS21, and FS28 (*n* = 3 per group) were crushed and sieved (Supplementary Materials S2). A 5 g portion of each powder was added to 250 mL of methanol, heated, and refluxed for 2 h to obtain the extract. After drying, 250 mL of distilled water was added, and reflux extraction was continued for an additional 1.5 h. The mixture was centrifuged at 3000 rpm for 5 min, and the 100 mL supernatant was combined with the methanol extract and freeze-dried to yield the total extract for each sample. A separate 100 mL methanol extract was distilled, dissolved in ultrapure water, and suspended. Total flavonoids and total saponins were then extracted from this suspension using ethyl acetate and n-butanol, respectively. The total polysaccharide samples from different fermentation times, as per Section 2.4.1, were collected and stored at 4 °C for future use.

#### 2.5.2. Establishment of the Cell Model

HK-2 cells were cultured in RPMI 1640 medium (RPMI 1640, Wuhan Pricella Biotechnology Co., Ltd., Wuhan, China) supplemented with 10% fetal bovine serum (FBS), 100 μg/mL streptomycin, and 100 units/mL penicillin. The cells were incubated at 37 °C in a humidified atmosphere containing 5% CO_2_, and the culture medium was refreshed every other day.

A total of 1.0 × 10^5^ HK-2 cells were plated in 24-well plates and divided into the control group, model group, and treatment groups (treated with total extracts from FS0, FS7, FS14, FS21, and FS28, respectively; *n* = 4 per group). After 24 h of culture, the control and model groups were replaced with fresh medium, while the positive groups were pre-incubated with 100 μmol/L allopurinol for 24 h. Following this, PBS was used to wash each well three times, and 2.5 mmol/L adenosine in serum-free medium was added to the model and positive groups. The control group was maintained in fresh medium without adenosine. After 30 h of incubation, 0.005 U/mg xanthine oxidase (XOD) was added to each well. The culture supernatant was collected 8 h post-treatment, and UA levels were measured by HPLC.

#### 2.5.3. Comparison of Uric Acid-Lowering Activity and Activity Monitoring

For quantification of UA content in the cell supernatant, HPLC-UV was performed based on the liquid-phase equipment conditions outlined in Section 2.4.1. A ZORBAX SB-Aq column (150 mm × 4.6 mm, 3.5 μm, AKZO NOBEL, Bohus, Sweden) was used as the stationary phase. The mobile phase comprised a 7 × 10^−3^ mol·L^−1^ KH_2_PO_4_-H_3_PO_4_ solution containing 10% acetonitrile (A) and a 7 × 10^−3^ mol·L^−1^ KH_2_PO_4_-H_3_PO_4_ solution (B). The gradient elution program was as follows: 0–6 min, 100% (B); 6–14 min, 100–30% (B); 14–17.4 min, 30% (B); 17.4–20 min, 30–100% (B); 20–30 min, 100% (B). UV absorption spectra were measured at a flow rate of 1.0 mL/min, a column temperature of 25 °C, and a wavelength of 283 nm.

#### 2.5.4. Efficacy Evaluation of Various Ingredients

A total of 1.0 × 10^5^ HK-2 cells were plated in 24-well plates and assigned to the following groups: control, model, total polysaccharide treatment, total flavonoid treatment, and total saponin treatment. The cells were cultured in 24-well plates for 24 h. After this, the control and model groups were switched to fresh medium, while the positive groups were pre-incubated with 100 μmol/L allopurinol for 24 h. Each well was then washed three times with PBS, and 2.5 mmol/L adenosine in serum-free medium was added to the model and positive groups. The control group was maintained in fresh medium without adenosine. After a 30 h incubation, 0.005 U/mg XOD was added to each well. The culture supernatant was collected 8 h after treatment, and the UA levels were measured by HPLC.

### 2.6. XOD Inhibition Assay

Firstly, a xanthine solution (1.5 mmol·L^−1^) was prepared by accurately weighing 22.8 mg of xanthine, dissolving it in a minimal volume of NaOH, and then diluting the solution to 100 mL with phosphate-buffered saline (PBS, pH 7.5) in a volumetric flask. Allopurinol, a known XOD inhibitor, was used as a positive control. A 100 µg·mL^−1^ allopurinol solution was prepared by dissolving 3 mg of allopurinol in 30 mL of DMSO. XOD was diluted with PBS to a working concentration of 0.2 U·mL^−1^ and freshly prepared before use. The administration of samples was carried out according to the procedure described in Section 2.5.3.

In each sample, the XOD inhibitory activity was assessed for the total extracts, total polysaccharide fractions, total flavonoid fractions, and total saponin fractions. A volume of 500 µL of each solution was mixed with 500 µL of XOD solution and subsequently combined with 3 mL of PBS buffer. The mixture was thoroughly agitated and then incubated at 25 °C for 3 h. Subsequently, 1 mL of the xanthine solution was added, rapidly mixed, and degassed by sonication for 5 s. The absorbance of each reaction mixture was immediately measured at 292 nm using a microplate reader, with PBS used as a blank control. The inhibition rate of XOD was calculated according to the following formula:

$$\mathrm{Inhibition}\;(\%)=\frac{A_{0}-A_{n}}{A_{0}}\times 100\%$$

where $A_{0}$ is the absorbance of the control group (without sample), and $A_{n}$ is the absorbance of the sample group.

### 2.7. Molecular Dynamics Simulation of Cycloastragenol-6-Glucoside with XOD

For further validation and to investigate the stability of the ligand-XOD complexes, MD simulations were performed. From the bioactive compounds identified in the fermentation products, cycloastragenol-6-glucoside, which exhibited the highest molecular docking score, was selected for MD simulations using the AMBER ff14SB force field within GROMACS. The AM1-BCC charges for the ligand were calculated using the Antechamber module in AmberTools. The GAFF2 and ff14SB force fields were used for the ligand and protein, respectively. The system was solvated in a cubic TIP3P water box, and K^+^/Cl^−^ ions were added to neutralize the system. Energy minimization was carried out using 2500 steps of steepest descent followed by 2500 steps of conjugate gradient. The system was heated from 0 K to 298.15 K over 200 ps under NVT conditions, followed by 500 ps of NVT equilibration and then 500 ps of NPT equilibration. A 100 ns production MD simulation was performed under NPT conditions with periodic boundary conditions. Non-bonded interactions were truncated at 10 Å, long-range electrostatic interactions were calculated using the Particle Mesh Ewald (PME) method, and hydrogen bonds were constrained using the SHAKE algorithm. Temperature was controlled using the Langevin thermostat with a collision frequency of 2 ps^−1^, and pressure was maintained at 1 atm. The integration time step was 2 fs, and trajectories were saved every 10 ps for analysis. Trajectory analyses, including root-mean-square deviation (RMSD), root-mean-square fluctuation (RMSF), and hydrogen bond analysis, was conducted to evaluate the stability of the complexes and the dynamic interactions between the ligand and active site residues.

### 2.8. Data Processing

Data acquisition and processing were performed using the Thermo Xcalibur 2.1 workstation. Statistical analysis was conducted with Dunnett’s multiple comparison test using GraphPad Prism, version 9.0 (GraphPad Software, San Diego, CA, USA). A *p*-value of <0.05 was considered statistically significant.

## 3. Results and Discussion

Hyperuricemia results from disrupted purine metabolism, leading to excessive UA accumulation, which contributes to conditions such as gout and metabolic disorders. UA, a byproduct of purine degradation, is primarily excreted by the kidneys. Effective management of hyperuricemia involves regulating UA levels and maintaining kidney function [35]. Common treatments include medications like phenylbromarone, allopurinol, and febuxostat. Additionally, TCM herbal constituents, including wolfberry and Dioscoreaceae rhizome saponins [36], may offer benefits by reducing inflammation and mitigating kidney damage. A study [20,37,38] in rats indicated that AR is more effective than benbromarone in managing hyperuricemia. Research is increasingly focused on the effects of Huaier-transformed AR on hyperuricemia. Univariate analysis of fermentation samples identified the 7th, 14th, 21st, and 28th days as critical points in the fermentation process (Supplementary Materials S1). In cell studies, the fermentation product of AR and Huaier demonstrated the most significant UA-lowering effects at 21 days of fermentation. Total flavonoids from AR and Huaier exhibited marked UA-lowering effects, peaking at day 7, and decreasing thereafter. Total saponins showed a progressive increase in UA-lowering effects, peaking at 21 days and declining by day 28. However, accurately monitoring component changes during AR fermentation to optimize the process remains challenging.

### 3.1. Monitoring of the Fermentation Process Based on Uric Acid-Lowering Activity Evaluation

Results of the efficacy evaluation of total extracts: Precise measurement of process parameters and quality attributes is crucial for optimizing biological processes. Mass spectrometry techniques, such as GC-MS and LC-MS, allow for sensitive, simultaneous quantification of multiple metabolites [39]. Metabolomics analysis of spent media can provide valuable insights into substrate uptake and metabolite secretion during AR fermentation [40]. These data help optimize feed ratios and fermentation durations. Although lacking real-time feedback, metabolomics remains a powerful tool in pharmacology for monitoring fermentation processes. Monitoring based on UA-lowering effects compensates for the absence of component-specific tracking. Analyzing metabolic changes during fermentation offers insights into the pharmacological effects of specific metabolites, highlighting the activation of biochemical pathways that drive therapeutic responses. These methodologies not only enhance the fermentation process but also shed light on emerging pharmacological effects.

**Figure 1:**
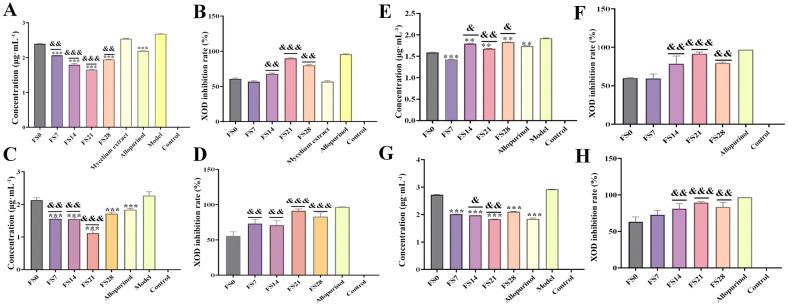
Results of uric acid-lowering efficacy and XOD inhibition rate test in each group. (**A**,**B**): Total extracts; (**C**,**D**): Total saponins; (**E**,**F**): Total flavonoids, (**G**,**H**): Total polysaccharides. (Each group was compared with the model group: ** *p* < 0.01, *** *p* < 0.001; Compared with the FS0 group: ^&^ *p* < 0.05, ^&&^ *p* < 0.01, ^&&&^ *p* < 0.001).

The relative biosafety of the total extract, total polysaccharides, total saponins, total flavonoids, and cycloastragenol-6-glucoside was demonstrated through in vitro cell safety experiments (Figure S1). Subsequent verification of the uric acid-lowering activity will be conducted within this safety interval. The fermentation product of AR and Huaier demonstrated the most significant UA-lowering effects at 21 days of fermentation (*p* < 0.001) (Figure 1A). Total saponins showed a progressive increase in UA-lowering effects, peaking at 21 days and declining by day 28 (*p* < 0.01) (Figure 1C). Total flavonoids from AR and Huaier exhibited marked UA-lowering effects, peaking at day 7, and decreasing thereafter (*p* < 0.001) (Figure 1E). As the most abundant active component in the fermentation product, polysaccharides demonstrated a significant uric acid-lowering effect by day 21 (*p* < 0.001) (Figure 1G). The total polysaccharide content of the fermentation product exhibited significant uric acid-lowering activity and a higher XOD inhibition rate (*p* < 0.001) (Figure 1H), with the most pronounced effect observed after 21 days of fermentation. These results highlight the importance of monitoring compositional changes during the fermentation of fungal substances to gain insights into pharmacodynamic indicators, particularly regarding the potent UA-lowering properties of polysaccharides and saponins.

Xanthine oxidase inhibitory activity of fermentation products: The XOD inhibitory activities of total extracts and fractions from different fermentation time points were evaluated. As shown in Figure 1B, all samples exhibited concentration-dependent XOD inhibition. Beyond the cellular models, the direct inhibitory effect of AR-Huaier fermentation products on XOD was investigated, as XOD is a pivotal enzyme in the purine catabolism pathway, directly responsible for uric acid production. Various fermentation stages and isolated components exhibited differential XOD inhibitory activities. Specifically, the total extracts from FS21 demonstrated the highest XOD inhibition rate of 86%, significantly surpassing that of FS0 (57%) and comparable to the positive control allopurinol (96%).

These findings are consistent with previous research highlighting the role of natural products, particularly flavonoids and certain saponins, in modulating XOD activity (Figure 1D,F). The observed increase in XOD inhibitory potential during fermentation, especially peaking around day 21, aligns with the enhanced hypouricemic effects seen in the HK-2 cell model. This suggests that the fermentation process not only transforms existing compounds but also potentially generates novel metabolites or increases the bioavailability of known inhibitors, thereby augmenting the overall XOD inhibitory capacity.

### 3.2. Spectrum–Effect Relationship Analysis

**Figure 2:**
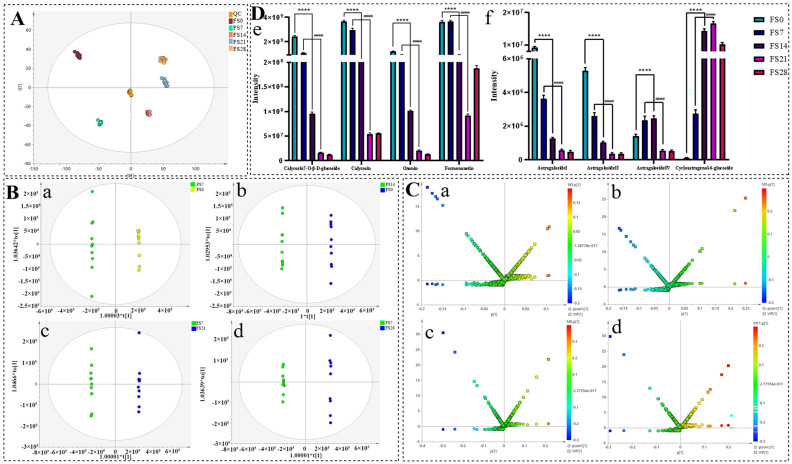
Metabolomics analysis results. ((**A**): PCA diagram of FS0, FS7, FS14, FS21, FS28 and QC samples; (**B**): OPLS-DA diagram of FS0, FS7, FS14, FS21 and FS28 samples; (**C**): S-plot + VIP plot of FS0, FS7, FS14, FS21 and FS28 samples (**D**): Significant metabolite relative content results (In the (**B**,**C**) diagram, (**a**): FS0 and FS7; (**b**): FS0 and FS14; (**c**): FS7 and FS21; (**d**): FS7 and FS28; In the (**D**) diagram, (**e**): Flavonoid differences; (**f**): Saponins differences; FS0 compared with FS14, **** *p* < 0.0001; FS7 Compared with FS21, ^####^ *p* < 0.0001).

Monitoring of metabolites after fermentation using metabolomics: Further analysis of the PCA results showed dense clustering of QC samples in Figure 2A, indicating high reproducibility and stability of the test instruments and acquisition system. To ensure scientific validity and robustness, OPLS-DA was employed for data monitoring. The robustness and predictive capability of the OPLS-DA model were evaluated by a permutation test (200 iterations). The intercepts for R^2^Y and Q^2^ were 0.253 and 0.0394, respectively, with *p* < 0.005, confirming that the model was not overfitted (Figure S8). As presented in Figure 2B,C, liquid chromatography-based analysis provides a solid foundation for assessing compositional transformations. A detailed examination of the FS0, FS7, FS14, FS21, and FS28 datasets revealed significant separation in metabolic profiles, reflecting the gradual alterations in AR composition during fermentation. This approach enables real-time monitoring of metabolic changes throughout fermentation, contributing to process optimization. For qualitative and quantitative metabolite evaluation, components were assessed and selected according to specific criteria, resulting in the identification of 299 potential metabolites. These metabolites were validated through liquid-phase analysis, qualitative data, compound standards, and existing literature. The analysis of 33 isolated metabolites, including 20 flavonoids and 13 saponins (Table 1), further elucidated the patterns of metabolite changes over fermentation time. By comparing the relative peak areas of metabolites among groups, a visual representation was provided to guide optimization of the fermentation strategy to maximize beneficial components for UA-lowering activity. Notably, eight components exhibited significant content changes (*p* < 0.0001) upon comparison with standards (Figure 1D), warranting further investigation into the implications of these changes for refining the fermentation strategy.

**Figure 3:**
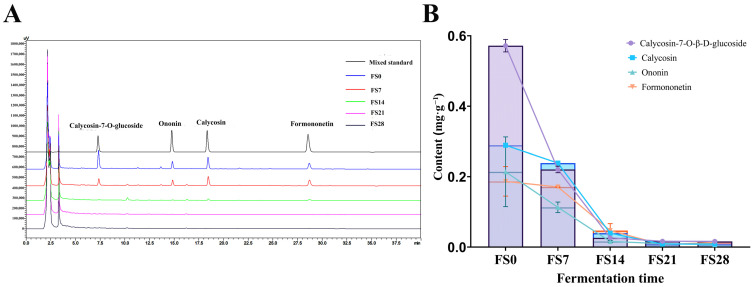
Map of variation in flavonoid content. ((**A**): chromatogram; (**B**): Change trend line chart of Calycosin-7-O-glucoside, Ononin, Calycosin, and Formononetin).

**Figure 4:**
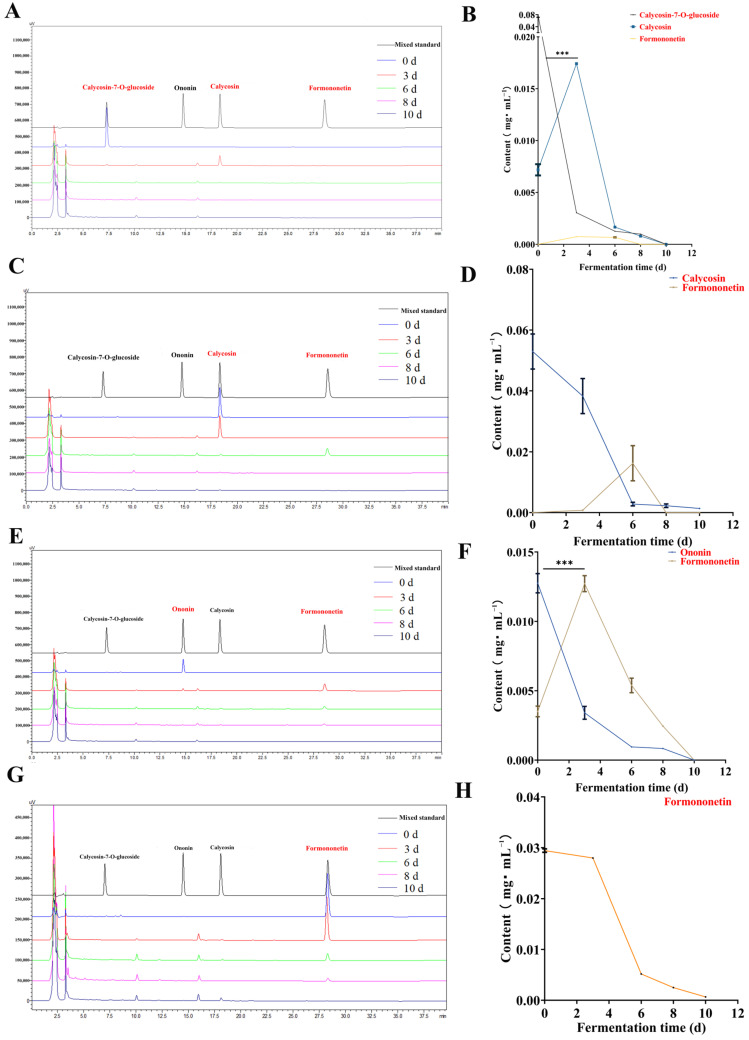
Trend diagram of fermentation products of each component. (**A**,**B**): Calycosin-7-glucoside, formononetin and calycosin; (**C**,**D**): Calycosin and formononetin; (**E**,**F**): Ononin and formononetin; (**G**,**H**): Formononetin (Compared with 3 d, *** *p* < 0.001).

Fermentation transformation analysis results of flavonoids and saponin compounds: Previous research has demonstrated that flavonoids inhibit XOD enzyme activity, thereby reducing UA levels and the progression of UA nephropathy. Fermentation can modify these flavonoids, converting calycosin-7-glucoside into calycosin within 3 days, which then transforms into formononetin. Calycosin and formononetin levels decline rapidly by day 6, with most being metabolized by day 8. Ononin also converts into formononetin within 3 days, depleting by day 10 (Figure 3). The content of formononetin notably decreases between days 3 and 6, with a gradual decline continuing until day 10 (Figure 4). Flavonoids transform during fermentation, as illustrated in Figure 1, where activity does not uniformly decrease. This suggests that new active compounds may emerge during fermentation, and the reduction in flavonoid levels could enhance the efficacy of the fermentation product.

**Figure 5:**
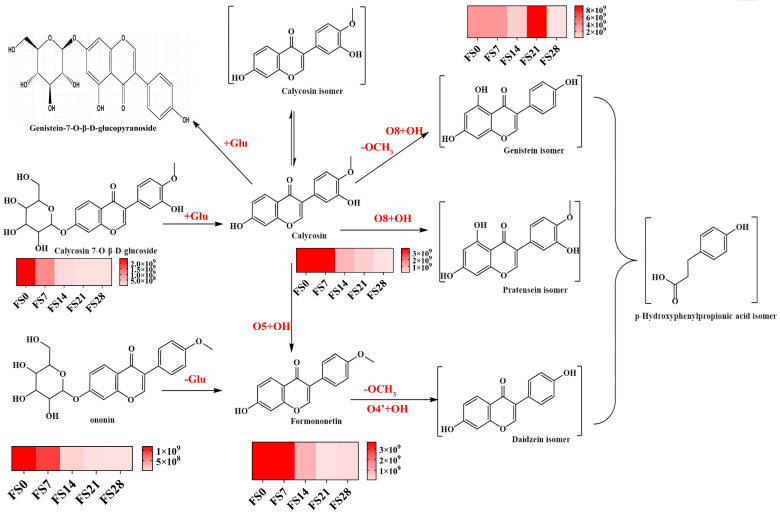
Fermentation and transformation pathways of flavonoids. (The red text indicates the cleavage-associated loss of functional group).

**Figure 6:**
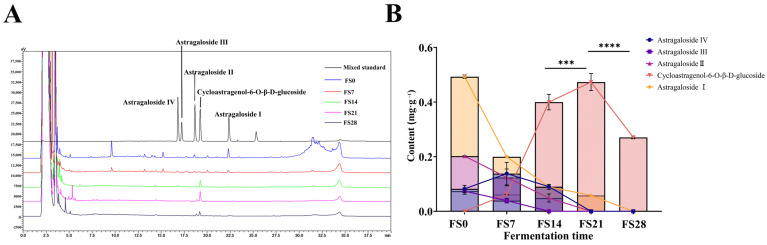
Map of variation in saponin content. ((**A**): chromatogram; (**B**): Change trend line chart of Astragaloside I, Astragaloside II, astragaloside III, Astragaloside IV, Cycloastragenol-6-glucoside) (Compared with fermentation product polysaccharides on day 21, *** *p* < 0.001, **** *p* < 0.0001).

The application of UHPLC Q-Exactive Orbitrap mass spectrometer was critical for detection and analysis, enabling dynamic monitoring of the fermentation process. This allows for the controlled transformation of flavonoid components, optimizing fermentation outcomes. When using UHPLC Q-Exactive Orbitrap mass spectrometry (Table 1, Figure 5), it was unexpectedly observed that primary non-sugar moieties or their isomers (calycosin, formononetin, genistein, daidzein) underwent mutual conversion after deglycosylation of flavonoid glycosides (Figure 6). These four aglycone constituents were detected in the fermentation samples of calycosin and formononetin. In the intermediate and later stages of fermentation, the flavonoid aglycones were further metabolized by the strain, producing p-hydroxyphenylpropionic acid derivatives. The decrease in certain components may not indicate a loss of efficacy but rather the emergence of novel UA-lowering activity, necessitating ongoing efficacy testing for real-time monitoring of the fermentation process.

**Figure 7:**
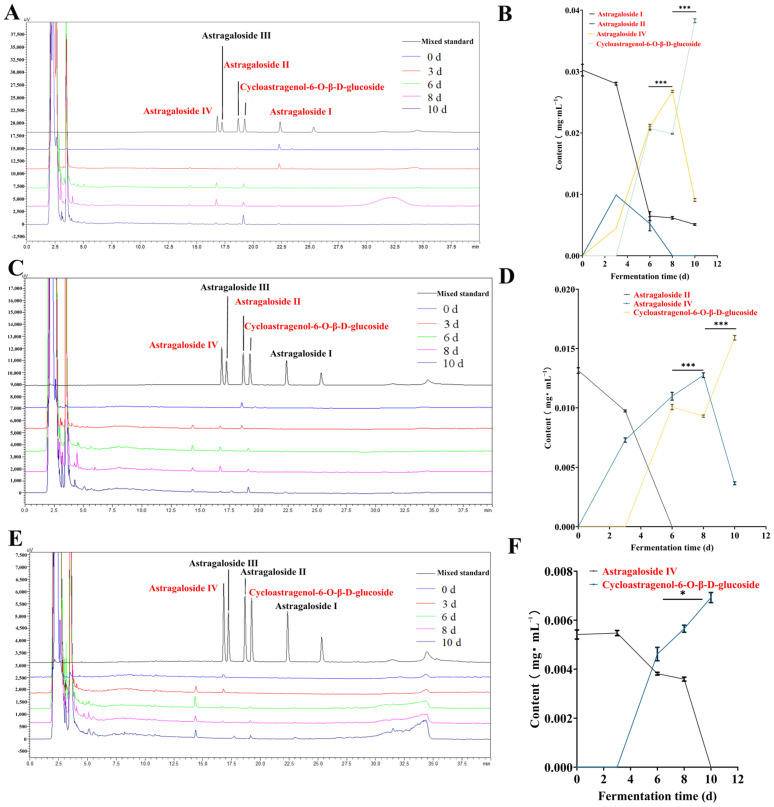
Trend diagram of fermentation products of each component. (**A**,**B**): Astragaloside I, astragalosides II and IV, cycloastragenol-6-glucoside; (**C**,**D**): Astragaloside II, astragalosides IV, and cycloastragenol-6-glucoside; (**E**,**F**): Astragaloside IV, and cycloastragenol-6-glucoside (Compared with 6 d, * *p* < 0.05, *** *p* < 0.001; Compared with 8 d, *** *p* < 0.001). During fermentation, the dynamics of saponins revealed the conversion of astragaloside IV to other astragalosides, thereby increasing the overall yield of astragaloside, which aligns with its role in reducing UA. UHPLC Q-Exactive Orbitrap mass spectrometry tracked the primary transformation mediated by sophorolysis-degrading enzymes. The main conversion pathway involved astragaloside I transforming into astragaloside II, which then converted to astragaloside IV, and ultimately astragaloside-6-glucoside. Additionally, minor reactions, such as dehydration, dehydrogenation, and ring cleavage, were observed, leading to dehydration products of astragaloside IV, dehydrogenation products of cycibaicalin-6-glucoside, and cleavage products of astragaloside II (Table 2, Figure 8).

In contrast to the pattern shown in Figure 7, the saponin components exhibited a rising and falling trend, possibly reflecting internal transformations and dominant utilization of certain saponins. During the fermentation of astragaloside I, astragalosides II and IV increased after an initial decrease over the first 3 days. Astragalosides I and II decreased significantly between days 3–6, while astragaloside IV and cycloastragenol-6-glucoside notably increased. The growth of astragaloside IV slowed from days 6–8, stabilizing with cycloastragenol-6-glucoside. After days 8–10, astragaloside IV declined rapidly, while cycloastragenol-6-glucoside increased. Astragaloside II decreased over the first 3 days, followed by an increase in astragaloside IV. Between days 3–6, astragaloside II sharply decreased, while astragaloside IV and cycloastragenol-6-glucoside increased. Astragaloside II converted to astragaloside IV from days 6–8, with a slight decrease in cycloastragenol-6-glucoside. After days 8–10, astragaloside IV decreased rapidly, while cycloastragenol-6-glucoside continued to increase. Astragaloside IV content slightly increased during the first 3 days, likely due to conversion from astragalosides I and II, followed by a steady decrease from days 3–10 as cycloastragenol-6-glucoside increased.

**Table 1:** Differential component identification results and data of fermentation products—Flavonoids and transformation products.

NO	t_R_ (min)	Molecular Formula	Theoretical *m*/*z*	Experiment *m*/*z*	Error/ppm	Fragment Ions	Metabolites
1	6.87	C_21_H_20_O_9_	417.11801	417.11771	−0.716	255.06(100.00), 227.07(4), 137.02(6), 119.05(1)	Daidzin/isomer
2 *	8.23	C_22_H_22_O_10_	447.12857	447.129	0.954	285.08(100), 270.05(28), 253.05(11), 225.05(8), 137.02(6)	Calycosin 7-O-β-D-glucoside ^△^
3	8.67	C_21_H_20_O_10_	433.11292	433.11285	−0.169	271.06(100), 253.05(2), 153.02(5), 119.05(1)	Genistin/isomer
4	9.63	C_21_H_20_O_10_	433.11292	433.11249	−1	271.06(100), 253.05(2), 153.02(8), 85.03(41)	Genistin/isomer
5	9.71	C_15_H_10_O_5_	271.0601	271.05988	−0.811	271.06(2), 253.05(5), 153.02(67), 121.03(12), 119.05(15), 91.05(100)	Genistein/isomer
6	10.16	C_22_H_22_O_11_	463.12317	463.12335	−0.298	286.05(100), 269.04(21), 241.05(9), 153.02(6)	Pratensein-glucoside/isomer
7	10.93	C_15_H_10_O_4_	255.06519	255.06503	−0.609	255.07(15), 227.07(5), 213.06(2), 145.03(94), 137.02(100), 119.05(47)	daidzein/isomer
8	11.31	C_15_H_10_O_5_	271.0601	271.05972	−1.402	271.06(16), 253.05(5), 153.02(56), 119.05(17), 91.05(100)	Genistein/isomer
9 *	11.31	C_15_H_10_O_4_	255.06519	255.06483	−1.393	237.05(3), 227.07(6), 137.02(68), 119.05(12)	daidzein/isomer
10 *	11.39	C_22_H_22_O_9_	431.13366	431.13379	0.305	269.08(100), 254.06(7), 237.05(3), 213.09(4), 137.02(1), 118.04(1)	Ononin ^△^
11	11.96	C_16_H_12_O_5_	285.07575	285.07587	0.421	270.05(13), 253.05(29), 213.05(100), 137.02(75), 134.04(76), 108.02(5)	Calycosin ^△^
12	11.98	C_9_H_10_O_3_	167.07027	167.07016	−0.663	151.04(3), 123.04(6), 121.03(5), 105.03(26), 95.05(62), 78.05(100)	p-Hydroxyphenylpropionic acid/isomer
13	12.29	C_9_H_10_O_3_	167.07027	167.07022	−0.304	151.04(2), 123.04(6), 121.03(4), 105.03(28), 95.05(58), 78.05(100)	p-Hydroxyphenylpropionic acid/isomer
14	13.46	C_16_H_12_O_6_	301.07066	301.07043	−0.779	286.05(16), 269.04(17), 241.05(44), 229.05(100), 153.02(82), 134.04(85)	Pratensein/isomer
15	13.46	C_16_H_12_O_5_	285.07575	285.07532	−1.508	285.08(9), 270.05(11), 253.05(16), 213.05(100), 137.02(4)	Calycosin/isomer
16	14.58	C_16_H_12_O_4_	269.08084	269.08087	0.129	269.08(6), 253.05(44), 237.06(28), 225.05(37), 197.06(100), 137.02(27), 118.04(47)	Formononetin ^△^
17 *	14.99	C_9_H_10_O_3_	167.07027	167.07021	−0.363	151.04(3), 123.04(7), 121.03(3), 105.03(22), 95.05(54), 78.05(100)	p-Hydroxyphenylpropionic acid/isomer
18	15.39	C_9_H_10_O_3_	167.07027	167.07002	−1.501	151.04(3), 123.04(5), 121.03(4), 105.03(29), 95.05(64), 78.05(100)	p-Hydroxyphenylpropionic acid/isomer
19	17.1	C_16_H_12_O_5_	285.07575	285.07529	−1.614	285.08(10), 270.05(13), 253.05(16), 213.05(100), 153.02(32)	Calycosin/isomer ^△^
20	21.02	C_16_H_12_O_4_	269.08084	269.08014	−2.584	269.08(3), 253.05(44), 237.06(27), 225.05(38), 197.06(100), 137.02(24), 118.04(58)	Formononetin/isomer

Note: “*” A compound that has been compared with a reference; “^△^” Potential metabolites with XOD inhibitory function screened by computer simulation analysis.

**Table 2:** Differential component identification results and data of fermentation products—Saponins and their transformation products.

NO	t_R_ (min)	Molecular Formula	Theoretical *m*/*z*	Experiment *m*/*z*	Error/ppm	Fragment Ions	Metabolites
1	13.98	C_41_H_68_O_14_	785.46818	785.46753	−0.831	455.35(16), 437.34(38), 419.33(17), 143.11(100), 125.10(17)	aIsostragaloside IV/isomer
2	15.49	C_41_H_66_O_14_	783.45253	783.45184	−0.885	471.35(3), 453.34(62), 435.33(100), 417.32(28), 201.16(33)	Dehydrogenation of Astragaloside IV
3 *	15.58	C_41_H_68_O_14_	785.46818	785.46582	−3.008	455.35(15), 437.34(30), 329.25(9), 143.11(100)	Astragaloside IV ^△^
4 *	15.77	C_41_H_68_O_14_	785.46818	785.4679	−0.36	473.36(11), 455.35(21), 437.34(26), 311.24(2), 143.11(100)	Astragaloside III ^△^
5	15.81	C_41_H_64_O_13_	765.44197	765.44122	−0.978	453.34(45), 435.33(100), 417.32(28), 201.16(49)	Dehydrogenation and dehydration of Astragaloside IV
6	16.29	C_36_H_56_O_9_	633.39971	633.39899	−1.136	471.35(8), 453.34(17), 435.33(42), 417.32(20), 201.16(100)	Dehydrogenation and dehydration of Cycloastragenol-6-O-β-D-glucoside
7	16.59	C_36_H_58_O_10_	651.41027	651.40961	−1.02	471.35(32), 453.34(24), 435.33(31), 417.32(26), 201.16(100)	Dehydrogenation of Cycloastragenol-6-O-β-D-glucoside
8 *	16.76	C_43_H_70_O_15_	827.47875	827.47791	−1.012	437.34(15), 175(81), 157(760), 143.11(100), 115.07(38)	Astragaloside II ^△^
9 *	16.9	C_36_H_60_O_10_	653.42592	653.42548	−0.68	455.35(18), 437.34(21), 238(31), 143.11(100), 125.10(33)	Cycloastragenol-6-O-β-D-glucoside ^△^
10	17.37	C_48_H_78_O_18_	943.52609	943.52563	−0.489	441.37(100), 423.36(62.50), 163.06(15.60), 141.02(57.41)	Soyasaponin Bb/isomer
11 *	19.41	C_45_H_72_O_16_	869.48931	869.48828	−1.187	455.35(8), 437.34(14), 217(56), 157(93), 143.11(100)	Astragaloside I ^△^
12	20.54	C_45_H_72_O_16_	869.48931	869.48816	−1.325	653.41(10), 455.35(13), 437.34(33), 419.33(21), 143.11(100.00)	Isoastragaloside I/isomer
13	26.26	C_37_H_60_O_13_	713.41067	713.40778	−4.048	437.34(5), 419.33(6)	F-ring cleavage of Astragaloside II

Note: “*” A compound that has been compared with a reference; “^△^” Potential metabolites with XOD inhibitory function screened by computer simulation analysis.

**Figure 8:**
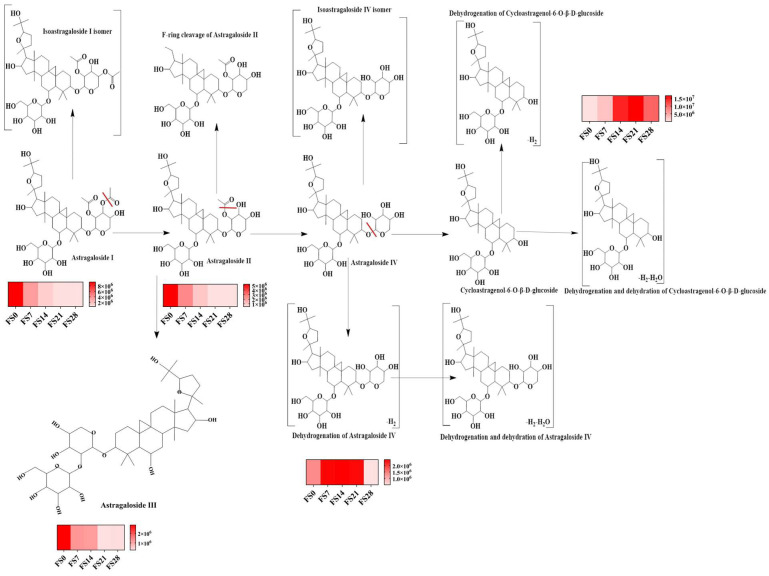
Fermentation transformation pathways of saponins. (The red line indicates the cleavage site).

Based on metabolic analysis, the “amount” changes in flavonoids and saponins in the fermentation process were determined: Single liquid metabolomics monitoring alone lacks the scientific rigor and data interaction verification necessary for comprehensive analysis. A more diversified monitoring approach facilitates visual analysis of the fermentation process and enables targeted analysis of key components. The HPLC method, based on the Chinese Pharmacopoeia (2025), was employed to analyze four flavonoids in fungal substances. Calycosin-7-glucoside and ononin eluted at 7.2 min and 14.7 min, while calycosin and formononetin eluted at 18.3 min and 28.6 min, respectively. During fermentation, the content of these flavonoids decreased significantly, with reductions observed after 28 days. Calycosin-7-glucoside dropped from 0.569 mg·g^−1^ to 0.016 mg·g^−1^, calycosin from 0.291 mg·g^−1^ to 0.009 mg·g^−1^, ononin from 0.137 mg·g^−1^ to 0.009 mg·g^−1^, and formononetin from 0.170 mg·g^−1^ to 0.010 mg·g^−1^. By the end of fermentation, the flavonoids had been almost entirely transformed, with their final contents remaining below 0.02 mg·g^−1^ (Supplementary Materials S2).

The fermentation process involves the degradation and conversion of active ingredients, with higher concentrations of free flavonoids associated with enhanced efficacy in reducing UA levels. In vitro studies assessing XOD inhibition have shown that the saponin extract exhibits the highest inhibitory activity, followed by the flavonoid extract. This highlights the significant role of saponin conversion during fermentation in enhancing UA-lowering activity. Consequently, careful monitoring of the fermentation process, optimization of fermentation conditions, and regulation of fermentation duration are essential. HPLC analysis revealed the levels of astragaloside I, II, IV, and cycloastragenol-6-glucoside in multiple fungal substance batches (Table 3). Astragaloside I exhibited a decreasing trend throughout fermentation, with its initial concentration of 0.493 mg·g^−1^ becoming nearly undetectable. Astragaloside II similarly decreased and was undetectable after 21 days of fermentation, while astragaloside IV also declined over the fermentation period. These results align with metabolomics data, providing complementary insights into the dynamics of saponin transformation during fermentation (Table 4).

**Figure 9:**
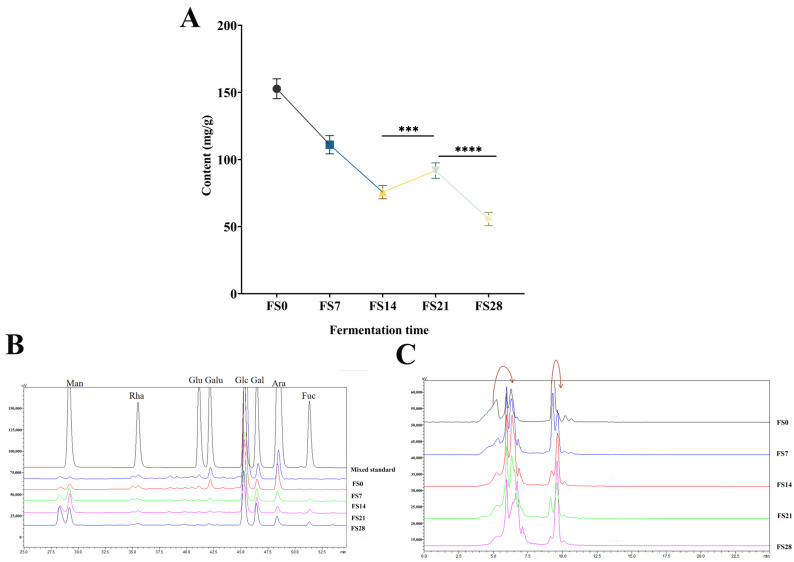
The composition of polysaccharides changes during fermentation. ((**A**): Changes in total polysaccharide content during fermentation; (**B**): Chromatographic diagram of monosaccharide composition changes in polysaccharides during fermentation; (**C**): Chromatographic diagram of molecular weight distribution of polysaccharides during fermentation. Compared to the fermentation product polysaccharide on day 21, *** *p* < 0.001, **** *p* < 0.0001) (Man: Mannose; Rha: Rhamnose monohydrate; Glu: Glucuronic acid; Galu: Galacturonic acid; Glc: Glucose; Gal: Galactose; Ara: Arabinose; Fuc: Fucose).

Quantitative analysis of polysaccharides in fungal substance samples: The detection method of total polysaccharide content is scientific and reliable (Supplementary Materials S3). As presented in Figure 9A,B, the peak metabolism of *Astragalus* polysaccharides occurred on the 14th day post-fermentation, followed by a notable decline. A transient increase was observed on the 21st day, which was then succeeded by a continuous decrease until the 28th day (Table 5). This pattern is likely due to the degradation and solubilization of cellulose in the plant material during fermentation, which serves as a substrate for fungal proliferation. As fermentation progressed, the levels of mannose and galactose continued to rise, while the glucose content decreased [22,29]. Additionally, the levels of glucuronic acid and galacturonic acid diminished significantly in the later stages of fermentation. Mycelium-specific fucose was detected after 7 days of fermentation and showed an increase throughout the fermentation process. A comparison of the chromatograms of astragalus and the mycoplasm after 7, 14, 21, and 28 days of fermentation (Figure 9C) revealed that the molecular weight distribution of astragalus polysaccharides gradually shifted towards smaller molecular weights as fermentation progressed. This suggests that hydrolytic enzymes and other substances produced by the strains have a certain decomposing effect on astragalus polysaccharides. At the same time, this process facilitates the breakdown of heteropolysaccharides, commonly present in *Astragalus* polysaccharides. Enhancing the proportion of active polysaccharides and improving the extraction rate of homogeneous polysaccharides is advantageous for maximizing their efficacy.

### 3.3. Dynamic Monitoring of Fermentation Process Based on Metabolomics and Pharmacodynamic Verification

Metabolomics technology has broad applications in various scientific fields, including TCM [41], clinical disease diagnosis [3,18], environmental science [42,43], and toxicology. In plant research, the diverse array of metabolites synthesized by plants presents significant challenges in analysis and monitoring [44,45]. Given the substantial value of many plant metabolites in food, medicine, and industry, investigating plant metabolomics is essential for advancing society and improving human health [46,47]. In this study, a UHPLC-Q-Exactive Orbitrap mass spectrometer was used to perform a qualitative analysis of the chemical constituents of AR and fungal substances at various fermentation stages, highlighting significant alterations during the fermentation process. The analysis revealed distinct segregation patterns between AR and Huaier at different fermentation time points (days 0, 7, 14, 21, and 28). These findings were corroborated by metabolomics-driven assessments of component transformations, resulting in the development of a structured system to monitor the fermentation process [48].

### 3.4. Correlation Analysis Between Component Transformation and Uric Acid-Lowering Efficacy

**Figure 10:**
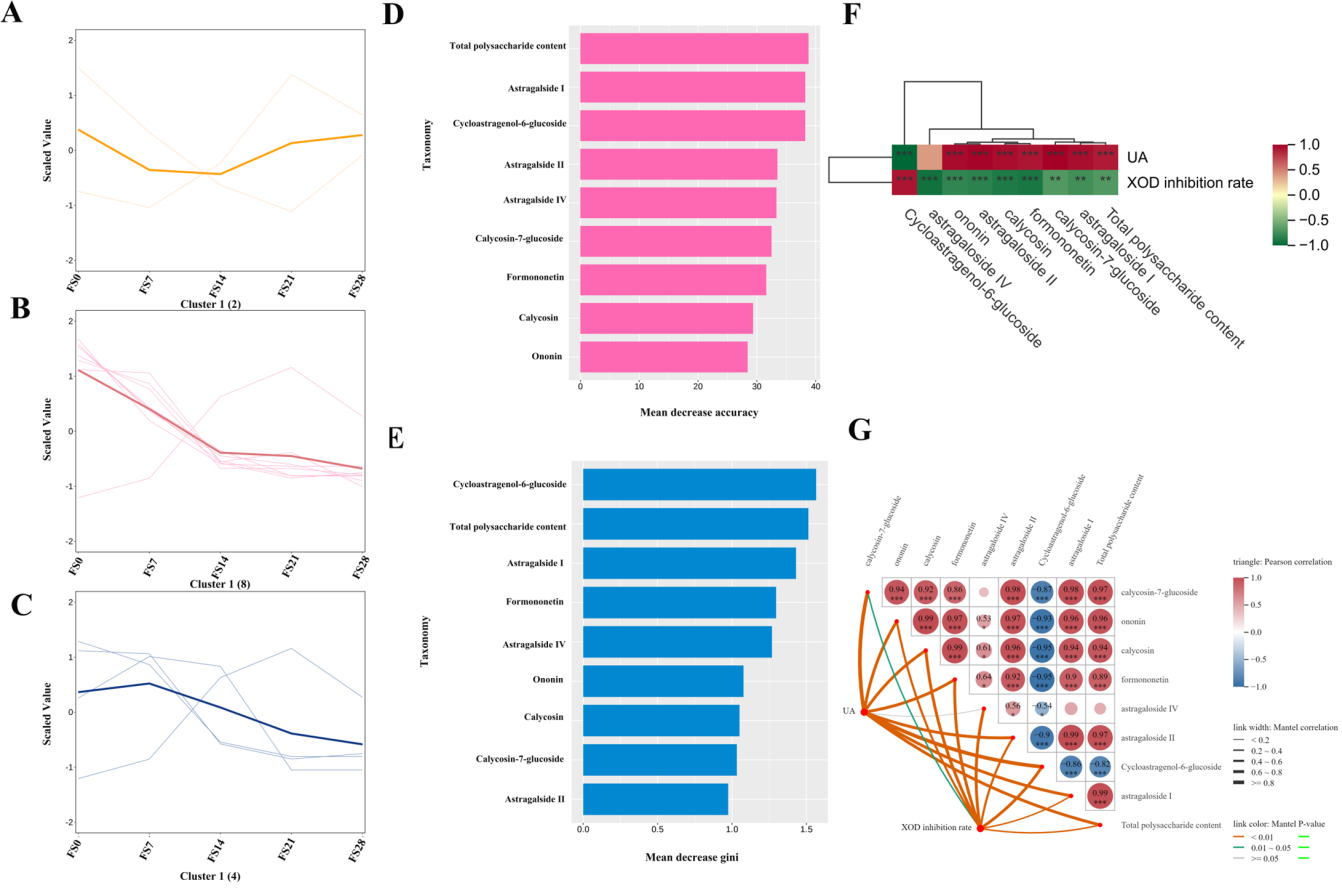
Spectrum–effect relationship analysis. (**A**–**C**): K-means correlation analysis of composition changes and efficacy at different fermentation times (Yellow: k-means map representing the efficacy results, red: k-means representing the expression level positively correlated with the changes of metabolomics components, blue: k-means representing the expression level negatively correlated with metabolomics). (**D**–**E**): Random forest diagram of differential metabolites and pharmacodynamic importance. (**F**): Correlation analysis of the effects of total flavonoids, saponins, and polysaccharide extracts on uric acid reduction. (**G**): Mantel test heatmap. (* *p* < 0.05, ** *p* < 0.01, *** *p* < 0.001).

By integrating metabolomics and pharmacodynamic data, this study elucidated a phased dynamic variation in chemical components throughout the fermentation process. Cycloastragenol-6-glucoside and total polysaccharide content reached a synergistic peak at 21 days of fermentation, coinciding with the lowest uric acid levels and the highest XOD inhibition rate. This observation indicates that these two components are key active constituents responsible for the uric acid-lowering effect observed in the bidirectional solid-state fermentation products of AR and Huaier. The continuous degradation of flavonoid glycosides and certain saponin aglycones may serve as precursor sources for the transformation into active aglycones. This pattern of “component fluctuation–activity synchronization” provides definitive chemical markers and scientific evidence for the precise control of the fermentation endpoint (21 days) and the development of functional foods with uric acid-reducing properties (Figure 10A–C). Total polysaccharides and Cycloastragenol-6-glucoside form the “dual-core” active signature, with the former impacting overall effects due to its dominant content, while the latter acts as the most sensitive biomarker through specific transformations and peak timing. The random forest model not only validated the component–activity correlations identified by metabolomics but also quantified the contribution of each feature, identifying polysaccharides and saponins as the primary active components in the fermentation products. This model provides robust statistical evidence for establishing day 21 as the optimal fermentation endpoint (Figure 10D,E).

The correlation analysis (Figure 10F,G) between the UA-lowering effects of total flavonoids, saponins, and polysaccharide extracts reveals that Cycloastragenol-6-glucoside is significantly positively correlated with both UA-lowering activity and XOD inhibition. This strong positive correlation, together with the peak content of this compound at day 21 (Figure 7E,F), coinciding with the highest hypouricemic activity (Figure 1), supports the conclusion that Cycloastragenol-6-glucoside is a key active component responsible for the UA-lowering effects of the fermented products [49]. To further quantify the relationship between individual metabolites and uric acid-lowering efficacy, we performed correlation analyses as shown in Figure S9. Figure S9A,B present a metabolism–pharmacodynamic correlation ring diagram and network. The ring diagram visually highlights that cycloastragenol-6-glucoside is centrally positioned, indicating its dominant role in UA reduction. The network analysis further reveals clusters of positively correlated flavonoids and saponins that collectively influence UA levels and XOD inhibition rate. Figure S9C–E display Pearson correlation and linear regression results for three key components. Figure S9C shows a strong negative correlation for cycloastragenol-6-glucoside (R = −0.949, *p* = 6.83 × 10^−8^), confirming its potent UA-lowering effect. Figure S9D reveals a strong positive correlation for calycosin-7-glucoside (R = 0.927, *p* = 6.62 × 10^−7^), indicating that its degradation during fermentation contributes to enhanced efficacy. Figure S9E similarly demonstrates a positive correlation for astragaloside II (R = 0.911, *p* = 2.38 × 10^−6^), further supporting that the decline of certain saponins is beneficial for UA reduction. These quantitative correlations provide robust statistical support for the spectrum–effect relationship and identify cycloastragenol-6-glucoside as a key biomarker candidate associated with the optimal fermentation endpoint (day 21).

This suggests that Cycloastragenol-6-glucoside may be the primary active compound responsible for the observed UA-lowering effects, with its highest concentration occurring on the 21st day of fermentation. The changes in flavonoids, saponins, and total polysaccharides in the fermented products exhibited a positive correlation with the uric acid-lowering activity. Notably, the total polysaccharide component, which had the highest content, demonstrated the most significant effect in reducing uric acid levels. This suggests that the fermentation process enhances the activity of uric acid reduction. Future research will focus on further exploring the active conversion components responsible for uric acid reduction to develop functional fermentation products. The fermentation technology applied to AR and Huaier effectively enhances the conversion of active ingredients that reduce UA levels.

### 3.5. Dynamics Simulation Insights into Hypouricemic Activity

This study’s findings underscore the critical need to employ untargeted metabolomics to monitor the dynamic transformation of metabolites during fermentation. Through random forest analysis and metabolomics correlation, the dominant active component, cycloastragenol-6-glucoside, was identified. To further elucidate the potential mechanisms underlying the observed hypouricemic activity, molecular docking and molecular dynamics simulations were used to investigate interactions between the key compounds identified and xanthine oxidase (XOD), thereby providing a theoretical basis for subsequent mechanistic experiments.

Given its highest binding affinity and strong XOD inhibitory activity, the cycloastragenol-6-glucoside–XOD complex was subjected to a 100 ns MD simulation to evaluate the stability of the binding mode (Figure S9A,B). The RMSD of the protein backbone stabilized at approximately 0.25 nm after 20 ns, indicating a stable complex (Figure S9C). The ligand RMSD relative to the protein remained below 0.3 nm throughout the simulation, suggesting stable binding. RMSF analysis showed that residues in the active site exhibited low fluctuations, with the ligand maintained consistent interactions (Figure S9D). The radius of gyration (Rg) and solvent-accessible surface area (SASA) remained relatively stable after the initial equilibration period, indicating a well-folded and compact protein structure (Figure S9E,F). The distance between the ligand and protein centers of mass remained stable during the simulation, confirming that the ligand remained bound within the binding pocket (Figure S9G). Hydrogen bond analysis revealed an average of 4–6 hydrogen bonds between the ligand and protein, with dynamic formation and breakage consistent with stable binding (Figure S9H).

Binding free energy decomposition indicated that several key residues, including LEU-331, LYS-340, and GLU-309, contributed favorably to ligand binding, primarily through electrostatic and hydrophobic interactions (Figure S9I). The free energy landscape (FEL) derived from RMSD and Rg revealed a single deep energy basin, indicating that the complex maintained a stable conformation throughout the simulation (Figure S9J,K). Electrostatic potential surface analysis showed that the binding pocket exhibited complementary positive and negative potentials, facilitating stable ligand binding (Figure S9L). Superimposition of structures at 0, 50, and 100 ns confirmed that the ligand remained stably bound, with only minor conformational adjustments observed (Figure S9M). MM-GBSA calculations yielded a binding free energy of −28.66 ± 4.04 kcal/mol (Table S27), with van der Waals and electrostatic energies as the major contributors, further confirming the strong binding affinity. These computational predictions are consistent with the observed XOD inhibitory activity and offer a plausible molecular rationale for the hypouricemic effects of the fermentation products. Further experimental validation is necessary to confirm the binding mode with xanthine oxidase (XOD) and to establish a clear mechanism of action. The integration of untargeted metabolomics with these advanced computational techniques offers a powerful strategy for identifying and validating bioactive compounds in complex natural product mixtures.

**Figure 11:**
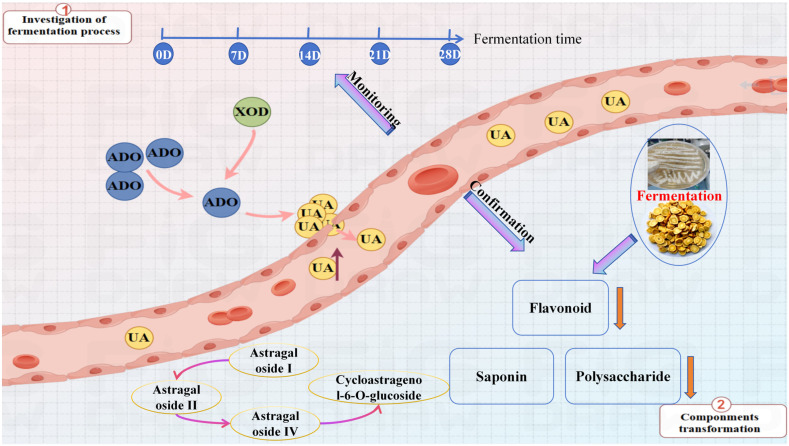
Process monitoring schematic diagram.

Figure 11 illustrates the internal dynamics of flavonoids, saponins, and polysaccharides during the fermentation process, as confirmed through dual verification *via* pharmacodynamic and metabolomics monitoring. This approach enabled accurate identification of changes in components over the fermentation period. Metabolomic analysis revealed significant reductions in calycosin-7-glucoside, ononin, calycosin, and formononetin from days 7 to 14. By day 14, calycosin-7-glucoside and ononin were nearly fully degraded, while calycosin and formononetin were almost completely degraded by day 21. No new flavonoids were detected throughout the fermentation period. The levels of astragaloside I and II consistently decreased over the 21 days, whereas astragaloside IV initially increased before declining. Cycloastragenol-6-glucoside levels rose during fermentation, peaking at day 21, followed by a subsequent decrease. The total polysaccharide content indicated that day 14 marked the peak of AR polysaccharide metabolism by Huaier, with a notable decrease thereafter. A brief increase in polysaccharide levels occurred by day 21 before continuing their decline through day 28.

Deglycosylation of flavonoid glycosides led to the transformation of four main aglycones—calycosin, formononetin, genistein, and daidzein—which were detected in the fermentation samples of calycosin and formononetin. Further enzymatic breakdown of these aglycones during fermentation produced p-hydroxyphenylpropionate derivatives. Despite the substantial metabolism of flavonoids and saponins, the UA-lowering effect increased progressively with fermentation time, with a significant turning point observed at day 21. The fermentation process induced significant metabolic alterations that directly influenced the formation of bioactive compounds and their associated UA-lowering activities. During the initial fermentation phase (days 0–21), the UA-lowering efficacy of total saponins and polysaccharides showed a positive correlation with fermentation duration. These findings are consistent with previous reports [50], suggesting that the combination of *Astragalus membranaceus* and Huaier produces a synergistic effect post-fermentation that is strongly correlated with enhanced UA-lowering activity. The present study did not include certain experimental controls, such as AR fermented without Huaier, Huaier alone, or a sterile process control. The inclusion of such controls would have allowed a more definitive attribution of compositional and functional changes to the specific interaction between AR and Huaier during fermentation. Therefore, while our results demonstrate a strong correlation between fermentation-induced chemical transformations and hypouricemic activity, the contribution of non-specific effects (e.g., thermal or oxidative degradation) cannot be completely excluded. Future studies will incorporate these additional controls to further validate the specificity of the observed effects and will focus on elucidating the mechanisms of the most potent fermentation-derived active ingredients in reducing UA, thereby advancing research on functional fermented foods. Consequently, a metabolomics-guided approach is critical to monitoring dynamic enzymatic activities throughout fermentation, enabling the optimization of UA-lowering compound production.

## 4. Conclusions

The purpose of this study was to explore the correlation between the chemical changes in the fermentation products of Astragalus and their implications for functional foods. This research aims to promote the development of functional foods that are homologous to medicinal products. This study employed metabolomics and pharmacodynamic monitoring to track changes in components during AR-Huaier fermentation and their effects on UA reduction. Metabolomics and HPLC analysis techniques were initially used to monitor fluctuations in flavonoids and saponins during fermentation. These analyses provided insights into the fermentation process, the metabolic pathways of key components, and established a reliable foundation for determining the optimal fermentation duration for maximizing the production of active substances that reduce UA levels. Subsequently, the impact of fermentation on UA reduction was evaluated using the HK-2 high UA cell model, revealing a strong correlation between the chemical profiles and the uric acid-lowering effects across different fermentation stages. This approach facilitated the optimization of fermentation strategies and identified the most effective fermentation conditions for lowering UA levels. Component transformation analysis revealed that flavonoids were predominantly metabolized by day 21 of fermentation, while astragaloside I, II, and IV were converted to cycloastragaloside-6-glucoside by the same time point. Polysaccharide levels stabilized after 14 days of fermentation, with a gradual increase in their UA-lowering activity. Among the monitored components, polysaccharides and saponins exhibited the strongest correlation with the reduction in uric acid (UA), indicating that they are the primary contributors to the hypouricemic activity. The study identified a 21-day fermentation period as optimal for UA reduction, with a decline in efficacy after 28 days due to fluctuations in cycloastragaloside-6-glucoside content. These findings offer a valuable correlation-based framework for investigating the fungal transformation of TCM components. A fermentation monitoring system was developed, focusing on “component transformation–pharmacodynamic” relationships, utilizing metabolomics to track fermentation progress, regulate component transformations, and ensure precise monitoring. This system establishes a foundation for understanding the mechanism of action of fermentation products with UA-lowering properties and aims to advance the development of fermentation-based therapeutic products for managing UA levels.

## Figures and Tables

**Table 3:** Results of flavonoid average content determination in samples. (*n* = 5, per group).

Fermentation Time	Calycosin-7-Glucoside (mg·g^−1^)	Ononin (mg·g^−1^)	Calycosin (mg·g^−1^)	Formononetin (mg·g^−1^)
FS0	0.569 ± 0.004	0.137 ± 0.015	0.291 ± 0.005	0.170 ± 0.019
FS7	0.216 ± 0.016	0.104 ± 0.024	0.237 ± 0.012	0.171 ± 0.007
FS14	0.026 ± 0.002	0.016 ± 0.0031	0.040 ± 0.008	0.031 ± 0.0018
FS21	0.016 ± 0.009	0.010 ± 0.006	0.009 ± 0.0016	0.007 ± 0.001
FS28	0.016 ± 0.0089	0.009 ± 0.0017	0.009 ± 0.0037	0.010 ± 0.009

**Table 4:** Results of the saponins’ average content determination in samples. (*n* = 5, per group).

Fermentation Time	Astragaloside IV (mg·g^−1^)	Astragaloside II (mg·g^−1^)	Cycloastragenol-6-Glucoside (mg·g^−1^)	Astragaloside I (mg·g^−1^)
FS0	0.067 ± 0.0074	0.202 ± 0.0174	0.000	0.493 ± 0.015
FS7	0.105 ± 0.0640	0.106 ± 0.0204	0.062 ± 0.0086	0.200 ± 0.036
FS14	0.090 ± 0.0081	0.039 ± 0.0174	0.337 ± 0.0174	0.089 ± 0.021
FS21	0.000	0.000	0.434 ± 0.0281	0.058 ± 0.017
FS28	0.000	0.000	0.271 ± 0.068	0.000 ± 0.004

**Table 5:** Changes in total polysaccharide content at different fermentation times. (*n* = 5, per group).

Fermentation Time	FS0	FS7	FS14	FS21	FS28
Average content (mg·g^−1^)	147.68 ± 11	107.64 ± 8	73.99 ± 9	90.03 ± 5	58.24 ± 7

## Data Availability

The original contributions presented in this study are included in the article/Supplementary Material. Further inquiries can be directed to the corresponding authors.

## Supplementary Materials

The following supporting information can be downloaded at: https://www.mdpi.com/article/10.3390/foods15101758/s1, Figure S1. Cytotoxicity of the extracts on HK-2 cells in vitro; Figure S2. Internal state of mycelium when the bag is full under different particle sizes; Figure S3. Internal state of mycelium at different temperatures when the bag is full; Figure S4. Internal state of different inoculation ratios when the bag is full of mycelium; Figure S5. Internal state of different water and material ratios when the bag is full of mycelium; Figure S6. Results of the linear relationship study for the glucose standard; Figure S7. Permutation plot of the OPLS-DA diagram; Figure S8. Metabolism-pharmacodynamic correlation analysis; Figure S9. Molecular dynamics analysis of the interaction between Cycloastragenol-6-O-β-D-glucoside and XOD. Supplementary Materials S1. Investigation on the Two-way Solid-state Fermentation Process of Astragali radix and Huaier (Tables S1–S10); Supplementary Materials S2. Ingredient Content Detection Methodology (HPLC) (Tables S11–S22); Supplementary Materials S3. Establishment of a Method for Determining Polysaccharide Content (Tables S23–S26); Table S27. Binding free energies and energy components predicted by MM/GBSA (kcal/mol).

## Abbreviation

AR: *Astragali radix*; TCM: Traditional Chinese Medicine; UA: Uric Acid; HRMS: High-Resolution Mass Spectrometry; UHPLC: Ultra-High-Performance Liquid Chromatography; HPLC: High-Performance Liquid Chromatography; ESI: Electrospray Ionization; PCA: Principal Component Analysis; OPLS-DA: Orthogonal Partial Least Squares Discriminant Analysis; VIP: Variable Importance in Projection; QC: Quality Control; FS0, FS7, FS14, FS21, FS28: Fermentation Samples (Day 0, 7, 14, 21, 28); ELSD: Evaporative Light-Scattering Detector; HK-2: Human Kidney 2 cells; PMP: 1-Phenyl-3-methyl-5-pyrazolone; LC-MS: Liquid Chromatography–Mass Spectrometry.
