# Supplementary material for: Chemical Components and Hypouricemic Activity Monitoring of Astragali radix-Huaier During Fermentation Processing Using High-Resolution Mass Spectrometry Combined with Untargeted Metabolomics
Source: Foods. 2026 May 15;15(10):1758. doi: 10.3390/foods15101758 (PMC13206624; doi:10.3390/foods15101758)
Supplement: Supplementary file 1 [file foods-15-01758-s001.zip › foods-4272492-supplementary.pdf]

Supplementary Materials

Figure S1. Cytotoxicity of the extracts on HK-2 cells *in vitro*.

Figure S2. Internal state of mycelium when the bag is full under different particle sizes.

Figure S3. Internal state of mycelium at different temperatures when the bag is full.

Figure S4. Internal state of different inoculated ratios when the bag is full of mycelium.

Figure S5. Internal state of different water and material ratios when the bag is full of mycelium.

Figure S6. Results of the linear relationship study for the glucose standard.

Figure S7. Permutation plot of the OPLS-DA diagram.

Figure S8. Metabolism-pharmacodynamic correlation analysis

Figure S9. Molecular dynamics analysis of the interaction between Cycloastragenol-6-O-β-D-glucoside and XOD.

Supplementary Materials S1. Investigation on the Two-way Solid-state Fermentation Process of *Astragali radix* and Huaier.

Supplementary Materials S2. Sample preparation and ingredient Content Detection Methodology (HPLC)

Supplementary Materials S3. Establishment of a Method for Determining Polysaccharide Content

Table S27. Binding free energies and energy components predicted by MM/GBSA (kcal/mol).

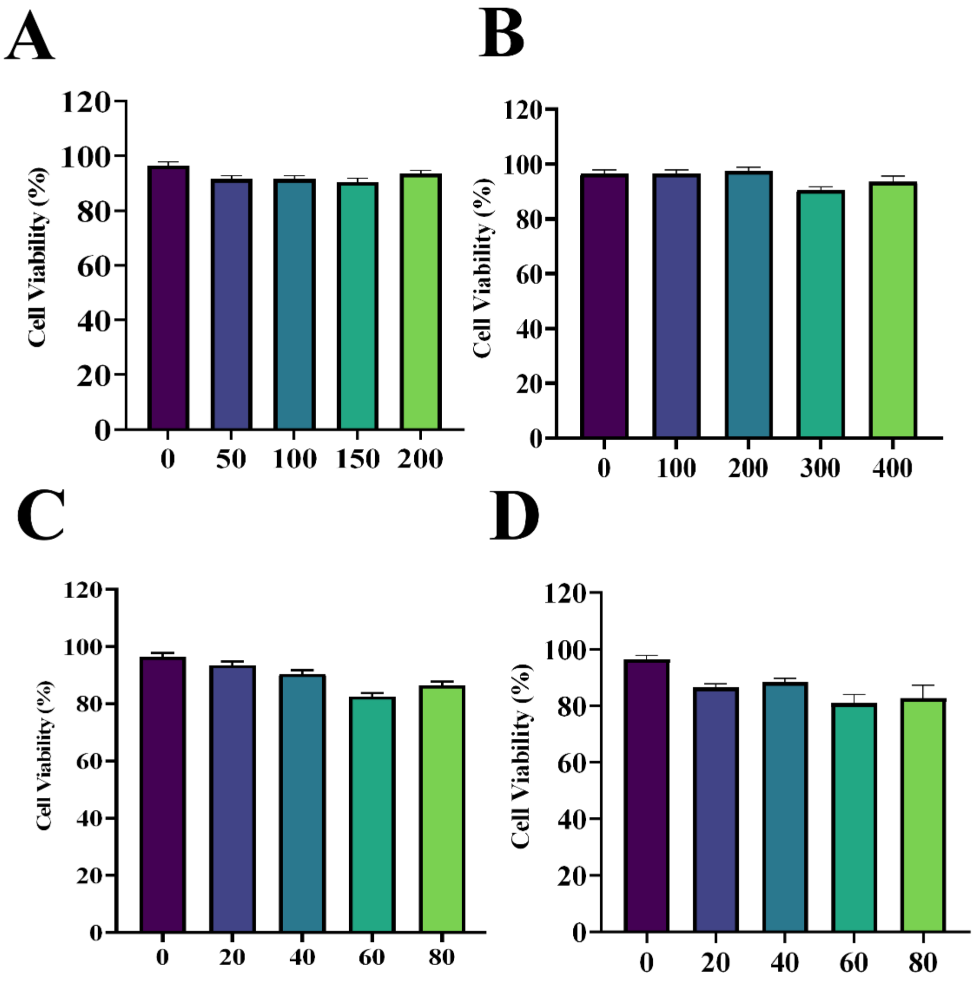

Figure S1. Cytotoxicity of the extracts on HK-2 cells in vitro. (A: The total extracts, B: The total polysaccharides, C: The total saponins, D: The total flavonoids).

S1. Investigation on the Two-way Solid-state Fermentation Process of Astragali radix and Huaier.

1.1. Strain activation and liquid culture

Under aseptic conditions, Huaier was inoculated onto potato dextrose agar (PDA) medium and incubated at 28 °C for 5 to 7 days to activate. Subsequently, an appropriate amount of the activated mycelium was collected with an inoculation loop and transferred into a sterilized potato dextrose liquid medium. This medium was then placed on a shaker at 28 °C and 150 r·min $^{-1}$ for 8 to 10 days to produce the seed liquid of Huaier.

1.2. Single-factor experiments

In the experiment, researchers initially employed the single-factor experiment method to examine the crushing particle size of *Astragali radix* slices, as well as the culture temperature, inoculation ratio, and water-to-material ratio during the cultivation process.

The study examined the particle size of *Astragali radix* by pulverizing and sieving it through 6-, 10-, 24-, and 50-mesh sieves. A 20 g portion of the sieved powder was placed in a fermentation bag, and 6 mL of distilled water was added. The bag was then sealed and autoclaved at 121 °C for 15 minutes. After cooling, 25 mL of Huaier seed liquid was introduced. Three samples were prepared in parallel and cultured at 28 °C until the hyphae completely covered the medium.

The investigation focused on fermentation temperature. *Astragali radix* was crushed and passed through a 10-mesh sieve. A 20 g portion of the sieved powder was placed in a fermentation bag, and 6 mL of distilled water was added. The bag was then sealed and sterilized using high-pressure steam at 121℃ for 15 minutes. After cooling, 25 mL of the seed solution was introduced. Three samples were prepared in parallel and cultured at 22℃, 25℃, 28℃, and 31℃, respectively, until the hyphae completely covered the medium.

In the investigation of the inoculation ratio, Astragali radix was crushed and passed through a 10-mesh sieve. A 20 g portion was placed in a fermentation bag, to which 6 mL of distilled water was added before sealing. The bag was autoclaved at 121℃ for 15 minutes and then allowed to cool. Three samples were prepared in parallel. Seed solutions of 15, 20, 25, and 30 mL were respectively added to these samples, which were then cultured at 28℃ until the mycelium completely covered the culture medium.

To examine the water-to-material ratio, the seed culture solution was excluded from the medium's total water content. *Astragali radix* was crushed and sifted through a 10-mesh sieve. Three parallel samples were prepared, with 20 g of each sample placed in a fermentation bag. Distilled water was added in volumes of 2, 6, 10, and 14 mL to each respective bag. The bags were then sealed and autoclaved at 121℃ for 15 minutes. After cooling, 25 mL of seed solution was added to each sample, which was then cultured at 28℃ until the mycelium fully covered the medium.

1.3. Orthogonal experiment

To optimize the fermentation process, I designed an L16(4^5) orthogonal experiment, focusing on temperature, crushing particle size, inoculation ratio, and water-to-material ratio as the key factors. The time required for mycelium to

fill the bag served as the evaluation index**. Table S1** presents the factors and levels for the fermentation process, while **Table S2** outlines the orthogonal experiment design scheme.

Table S1. Factors and levels of the fermentation process.

Number	Temperature (A).	Size (B).	Vaccination ratio (C).	Water-to-material ratio (D).
1	22℃	6-mesh sieve	75%	10%
2	25℃	10-mesh sieve	100%	30%
3	28℃	24-mesh sieve	125%	50%
4	31℃	50-mesh sieve	150%	70%

Table S2. Orthogonal experimental design scheme.

Number	Temperature (A)	Size (B)	Vaccination ratio (C)	Water-to-material ratio (D)	Blank (E)
1	1	1	1	1	1
2	1	2	2	2	2
3	1	3	3	3	3
4	1	4	4	4	4
5	2	1	2	3	4
6	2	2	1	4	3
7	2	3	4	1	2
8	2	4	3	2	1
9	3	1	3	4	2
10	3	2	4	3	1
11	3	3	1	2	4
12	3	4	2	1	3
13	4	1	4	2	3
14	4	2	3	1	4
15	4	3	2	4	1
16	4	4	1	3	2

1.4. Verification test

The fermentation of *Astragali radix* was conducted following the optimal process. The time when the mycelium filled the bags was recorded, and the growth state at that moment was observed. Simultaneously, these observations were compared with the fermentation conditions reported in the literature.

1.5. Results and Analysis

1.5.1. Results of single-factor investigation

1.5.1.1. Investigation of Medicinal Material Particle Size

Table S3 presents the time it took for mycelium to fill the bags when Astragali radix was crushed into various particle sizes, while **Figure S2** illustrates the internal state once the mycelium filled the bags. The results indicated that Huaier grew fastest in Astragalus powder that passed through a 10-mesh sieve,

with complete internal mycelium growth. It also grew relatively quickly in Astragalus powder that passed through a 6-mesh sieve, although the larger Astragalus particles resulted in incomplete internal mycelium growth. The growth rate and internal growth state of Huaier in Astragalus powder that passed through a 24-mesh sieve were inferior to those in the 10-mesh sieve powder. For the powder that passed through a 50-mesh sieve, the mycelium failed to fill the bags in the later growth stages, indicating poor growth. Thus, the Astragalus powder that passed through a 10-mesh sieve was deemed the optimal particle size for crushing Astragalus.

Table S3. Bag filling time of mycelium under different particle sizes.

Size	Full bag time (d).
6-mesh sieve	22
10-mesh sieve	21
24-mesh sieve	24
50-mesh sieve	-

Note: "-" means that the mycelium cannot fill the bag.

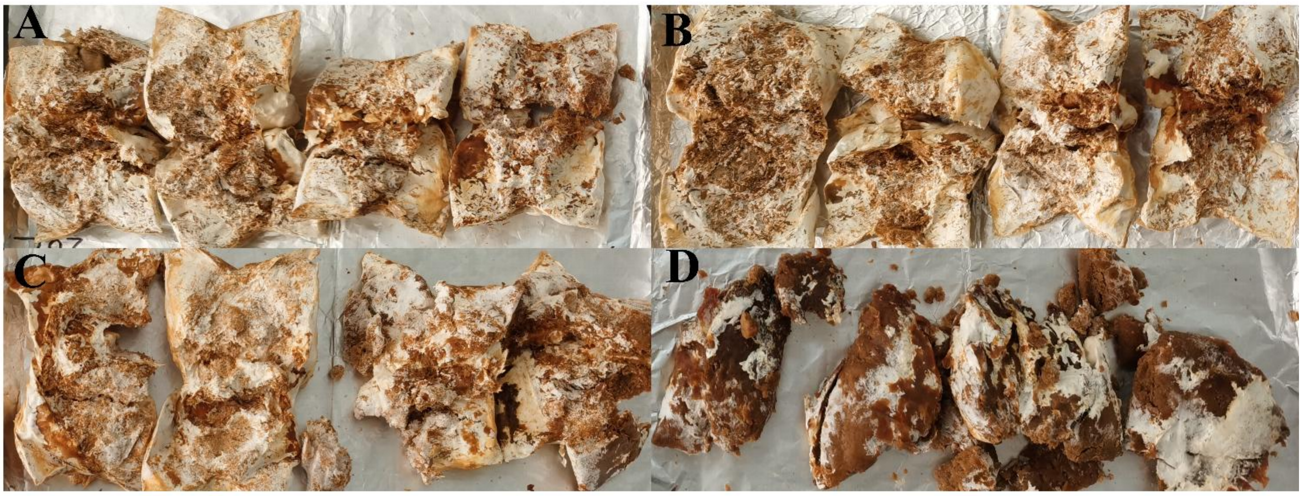

Figure S2. Internal state of mycelium when the bag is full under different particle sizes. (A: 6-mesh screen; B: 10-mesh screen; C: 24-mesh screen; D: 50-mesh screen).

1.5.1.2. Investigation of fermentation temperature

Table S4 display the time it took for the mycelium to fill the bags at various culture temperatures, while **Figure S3** illustrate the internal state once the mycelium filled the bags. The results indicated that at a culture temperature of 28°C, the mycelium grew the fastest, and the internal growth state was optimal when the bags were filled. At 31°C, the mycelium of Trametes robiniophila ceased growing after minimal development, suggesting that this temperature was unsuitable for its growth. Consequently, 28°C was selected as the optimal culture temperature for fermentation.

Table S4. Bag filling time of mycelium at different culture temperatures.

Temperature (°C).	Full bag time (d).
22	28
25	24

28	21
31	-

Note: “-” means that the mycelium cannot fill the bag.

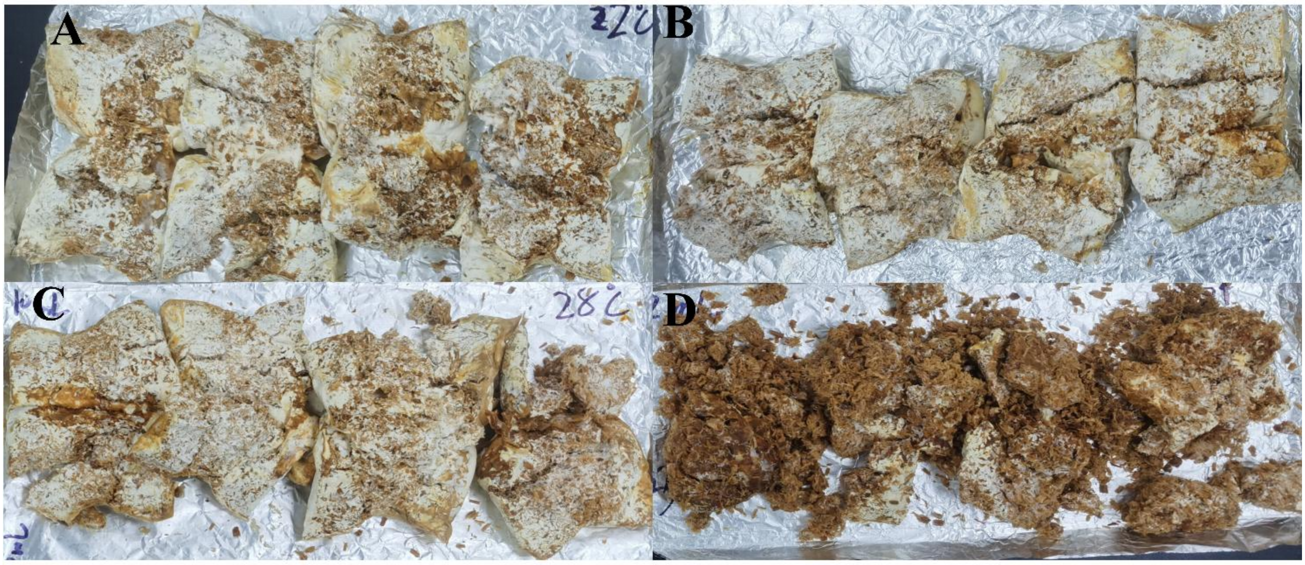

Figure S3. Internal state of mycelium at different temperatures when the bag is full. (A: 22℃; B: 25℃; C: 28℃; D: 31℃).

1.5.1.3. Investigation of the inoculation ratio of the seed liquid

Table S5 display the time required for the mycelium to fill the bags at various inoculation ratios, while **Figure S4** illustrate the internal state when the mycelium filled the bags. The results indicated that introducing a larger initial amount of mycelium was advantageous for its subsequent growth. Conversely, a minimal amount of mycelium hindered its growth. Taking inoculation efficiency into account, a 150% inoculation ratio was ultimately chosen as optimal.

Table S5. The filling time of mycelium at different inoculation ratios.

Vaccination ratio	Full bag time (d).
75%	-
100%	30
125%	23
150%	21

Note: “-” means that the mycelium cannot fill the bag.

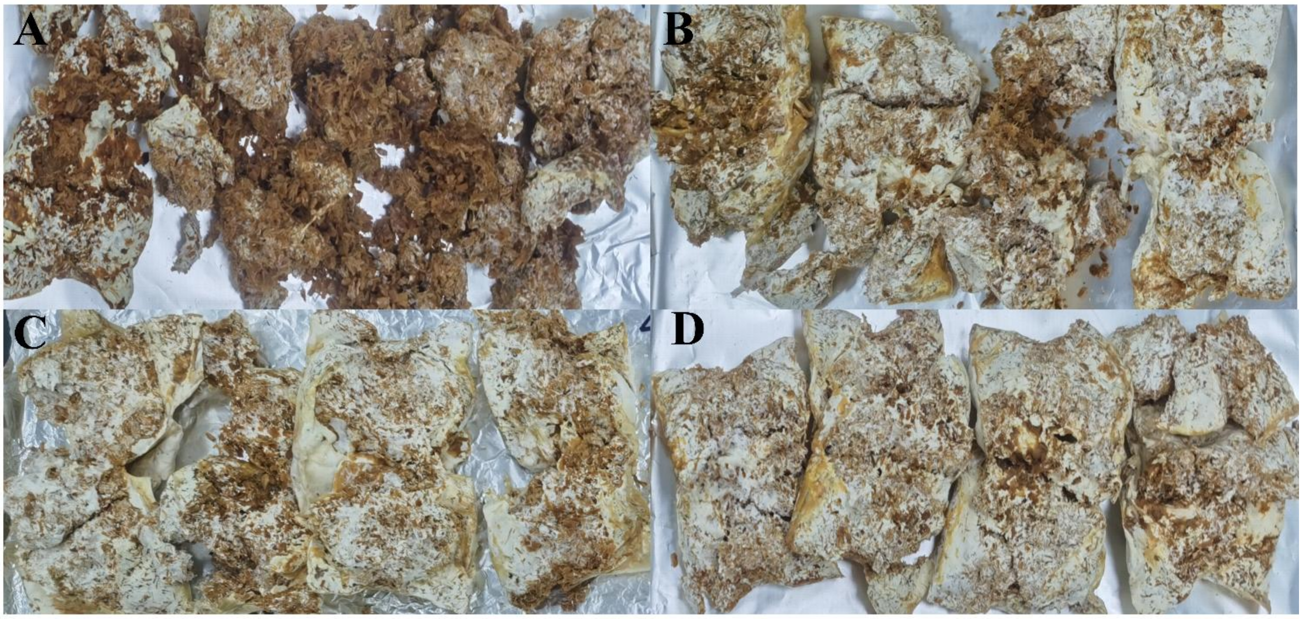

Figure S4. Internal state of different inoculated ratios when the bag is full of mycelium. (A: 75%; B:100%; C: 125%; D: 150%).

1.5.1.4. Investigation of the water-to-material ratio

Table S6 display the time needed for the mycelium to completely colonize the bags at various water-to-substrate ratios, while **Figure S5** illustrate the internal condition of the bags upon full colonization. The findings suggested that, up to a certain point, increasing the amount of water accelerated mycelium growth. However, when water was added excessively, the mycelium initially grew quickly, but later, the high water content and poor air permeability hindered its growth. Consequently, a water-to-substrate ratio of 50% was chosen as the optimal ratio.

Table S6. The filling time of mycelium with different ratios of water and material.

Water - material ratio	Full bag time (d).
10%	24
30%	23
50%	20
70%	18

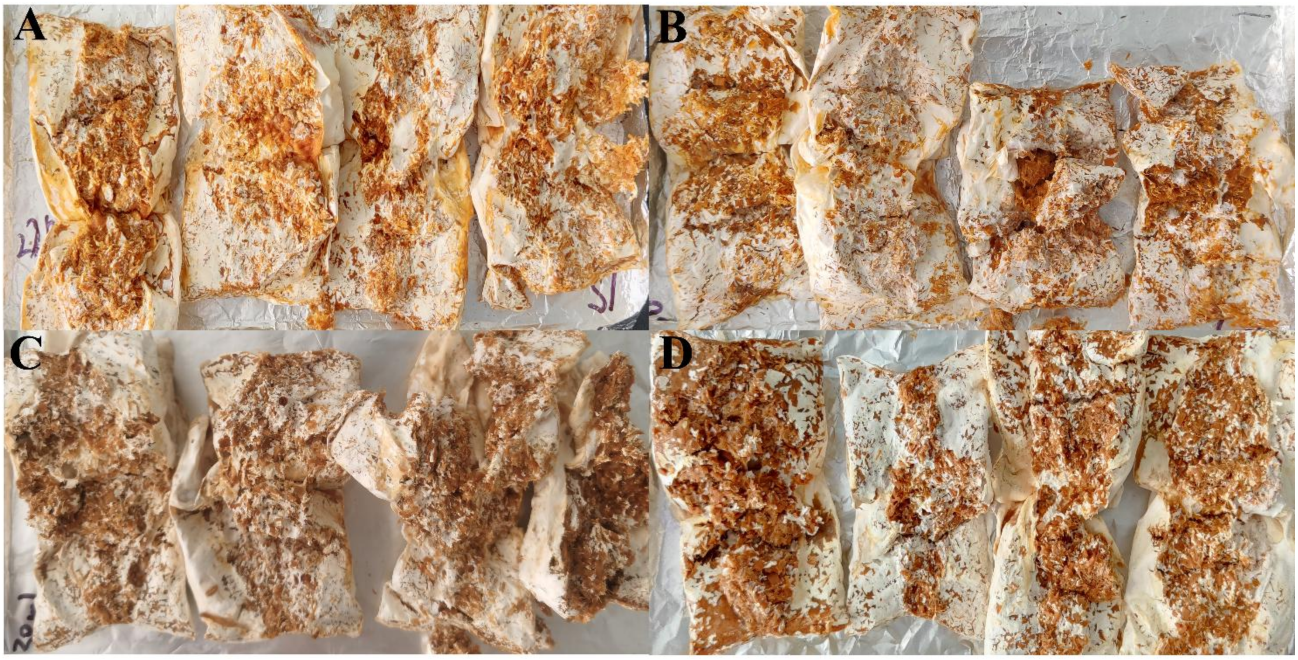

Figure S5. Internal state of different water and material ratios when the bag is full of mycelium. (Note: A: 10%; B:30%; C: 50%; D: 70%).

1.5.2. Results of orthogonal experiment

Range and variance analyses were performed on the results of the L16(4^5) orthogonal experiment. The range analysis results **(Table S7)** revealed that the factors ranked in order of significance were: Factor B (particle size) > Factor C (inoculation ratio) > Factor A (temperature) > Factor D (water–material ratio), with the optimal combination $A_3B_2C_4D_3$. The variance analysis results **(Table S8)** showed that particle size was a significant factor ($p < 0.05$). Observing the internal state of Astragali radix after the mycelium filled the bags confirmed $A_3B_2C_4D_3$ (cultivation at 28°C, Astragali radix crushed through a 10-mesh sieve, inoculation ratio of 150%, and water–material ratio of 50%) was indeed optimal. Consequently, the optimal fermentation process was determined to be cultivation at 28°C, Astragali radix crushed through a 10-mesh sieve, an inoculation ratio of 150%, and a water–material ratio of 50%.

Table S7. Range analysis results of the orthogonal test.

54	Temperatur e (A)	Size (B)	Vaccination ratio (C)	Water-to-material ratio (D)	Blank (E).	Full bag time (d).
1	1	1	1	1	1	100
2	1	2	2	2	2	41
3	1	3	3	3	3	37
4	1	4	4	4	4	100
5	2	1	2	3	4	30
6	2	2	1	4	3	35
7	2	3	4	1	2	32
8	2	4	3	2	1	100
9	3	1	3	4	2	23

Number	Temperature (A)	Size (B)	Vaccination ratio (C)	Water-to-material ratio (D)	Blank (E).	Full bag time (d).
10	3	2	4	3	1	20
11	3	3	1	2	4	41
12	3	4	2	1	3	100
13	4	1	4	2	3	28
14	4	2	3	1	4	33
15	4	3	2	4	1	41
16	4	4	1	3	2	100
K1	278	181	276	265	261	
K2	197	129	212	210	196	
K3	184	151	193	187	200	
K4	202	400	180	199	204	
k1	69.50	45.25	69.00	66.25	65.25	
k2	49.25	32.25	53.00	52.50	49.00	
k3	46.00	37.75	48.25	46.75	50.00	
k4	50.50	100.00	45.00	49.75	51.00	
R	23.5	67.75	24	19.5	16.25	

Note: For the convenience of calculation, the case of failure to grow mycelium or failure to fill the bag was calculated according to the time of filling the bag at day 100.

Table S8. Results of analysis of variance by orthogonal test.

Source of variance	Sum of squares of deviation	Degree of freedom	Mean square	F	P	Significance
A	1355.6875	3	451.896	1.921	0.303	>0.05
B	11718.1875	3	3906.063	16.605	0.023	<0.05
C	1359.6875	3	453.229	1.927	0.302	>0.05
D	891.1875	3	297.063	1.263	0.426	>0.05
E	705.6875	3	235.229	1.000	0.500	

1.5.3. Verification test results

The fermentation of *Astragali radix* followed the optimal process, and the time taken for the mycelium to fill the bags was recorded as 20 days, 20 days, and 21 days, respectively. The internal mycelium exhibited a healthy growth state. Compared to the process described in the literature, the mycelium grew faster and maintained a good internal condition, indicating the reliability of the optimized fermentation process. Additionally, the culture time was extended, and the fermentation process was documented. By the 7th day of fermentation, after a brief adaptation period, Trametes robiniophila began growing on *Astragali radix*, with some white mycelium appearing on the surface. By the 14th day, the mycelium grew rapidly, nearly enveloping *Astragali radix* externally, although it had not yet penetrated inside. By the 21st day, the mycelium had wrapped around *Astragali radix* and started to penetrate and grow internally, where they became thoroughly mixed with the *Astragali radix* powder. By the 28th day, T. robiniophila had largely consumed *Astragali radix,* causing the entire fungal substance to begin shriveling **(Table S9-10)**.

Table S9. Range analysis results of the orthogonal test.

Number	Temperature (A)	Size (B)	Vaccination ratio (C)	Water-to-material ratio (D)	Blank (E).	Full bag time (d).
1	1	1	1	1	1	100
2	1	2	2	2	2	41
3	1	3	3	3	3	37
4	1	4	4	4	4	100
5	2	1	2	3	4	30
6	2	2	1	4	3	35
7	2	3	4	1	2	32
8	2	4	3	2	1	100
9	3	1	3	4	2	23
10	3	2	4	3	1	20
11	3	3	1	2	4	41
12	3	4	2	1	3	100
13	4	1	4	2	3	28
14	4	2	3	1	4	33
15	4	3	2	4	1	41
16	4	4	1	3	2	100
K1	278	181	276	265	261	
K2	197	129	212	210	196	
K3	184	151	193	187	200	
K4	202	400	180	199	204	
k1	69.50	45.25	69.00	66.25	65.25	
k2	49.25	32.25	53.00	52.50	49.00	
k3	46.00	37.75	48.25	46.75	50.00	
k4	50.50	100.00	45.00	49.75	51.00	
R	23.5	67.75	24	19.5	16.25	

Note: For the convenience of calculation, the case of failure to grow mycelium or failure to fill the bag was calculated according to the time of filling the bag, 100 days.

Table S10. Results of analysis of variance by orthogonal test.

Source of variance	Sum of squares of deviation	Degree of freedom	Mean square	F	P	Significance
A	1355.6875	3	451.896	1.921	0.303	>0.05
B	11718.1875	3	3906.063	16.605	0.023	<0.05
C	1359.6875	3	453.229	1.927	0.302	>0.05
D	891.1875	3	297.063	1.263	0.426	>0.05
E	705.6875	3	235.229	1.000	0.500	

1.6. Discussion

Traditional Chinese medicine fermentation traditionally relied on naturally occurring environmental bacteria, which were typically mixed strains isolated without artificial intervention. This fermentation process involved a variety of strains in differing quantities, heavily influenced by environmental and seasonal changes. Due to its inherent variability and unpredictability, subjective empirical judgment was necessary for quality control. In contrast, modern traditional Chinese medicine fermentation employed a new process that strictly controlled

factors such as temperature, pH, humidity, and oxygen levels. It also regulated the quantity and type of fermentation bacteria to maintain environmental control throughout the process. This approach resulted in purer and more precise fermentation products, thereby enhancing product quality. Consequently, determining the optimal external conditions for microbial growth and ensuring their adaptation to the traditional Chinese medicine matrix remain a crucial foundational issue. Selecting the best fermentation process was essential for subsequent pharmacodynamic studies and matrix research. Currently, the primary process optimization indicators commonly chosen for fungal fermentation of traditional Chinese medicine include:

(1) Researchers selected component content as the preferred indicator, as component indicators were the most commonly used for process optimization. They used flavonoid content as the indicator and chose the solid-to-liquid ratio, inoculum size, fermentation time, and fermentation temperature as influencing factors. These factors were explored for their effects on flavonoid production during Houttuynia cordata fermentation, which led to the optimization of the fermentation process.

(2) Enzyme activity served as the optimization index. Using protease and amylase activities as indicators, the two-strain co-fermentation process of Liushenqu was optimized through a single-factor investigation and the Box–Behnken response surface design.

(3) Pharmacodynamic effects served as the preferred indicator. A single pharmacodynamic effect of the raw material drug was selected to optimize the fermentation process.

(4) Researchers selected mycelial growth as the preferred indicator. Mycelial growth served as the most intuitive measure for assessing fermentation processes. Indicators like the dry weight of mycelium, the growth rate of mycelium, and the growth state of mycelium were reported and utilized for evaluation.

Traditional process optimization typically relied on composition or a single index as the reference standard, often overlooking the mycelium's growth state during fermentation. Additionally, fermentation induced transformations in various components of medicinal materials, altering their contents. Thus, using components as indicators proved unscientific. The complexity of components in fermented fungal substances made it challenging to select representative and scientific indicators. During mycelium growth, various poor growth states could occur; however, an optimal growth state was achievable by controlling multi-factor and multi-level environments. In this chapter, the mycelium growth rate and growth state served as indicators to observe the effects of different environmental factors on mycelium growth most intuitively. This approach allowed for a preliminary and scientific optimization of the best fermentation process. Based on this optimized process, the fermentation endpoint was further determined by evaluating component transformation during fermentation and medicinal efficacy.

Through single-factor experiments and orthogonal tests, the optimal process was determined as follows: culturing at 28 °C, pulverizing Astragali radix and passing it through a 10-mesh sieve, with an inoculation ratio of 150% and a water-to-material ratio of 50%. During fermentation, on the 7th day, after several days of adaptation, Tremella fuciformis began to grow on Astragali radix, with a few hyphae appearing on the surface. By the 14th day, the mycelium grew rapidly,

nearly enveloping Astragali radix externally. On the 21st day, the mycelium penetrated and grew into the interior, completely mixing with the Astragali radix powder. By the 28th day, Tremella fuciformis had largely consumed Astragali radix, causing the fungal substance to begin shriveling. Based on the growth state of the mycelium at each time point, it was preliminarily predicted that the fermentation endpoint might be the 21st day.

S2 Sample preparation and ingredient Content Detection Methodology (HPLC)

1. Sequential solvent extraction procedure for flavonoids, saponins, and polysaccharides from fermented Astragali radix-Huaier samples

1.1. Fractionation protocol

Total flavonoids, total saponins, and crude polysaccharides were isolated from the non-fermented control (FS0) and fermented samples (FS7, FS14, FS21, FS28) using the following sequential extraction procedure.

1.2. Methanol extraction (total crude extract)

Sample powder (5 g) was extracted with methanol (250 mL) under reflux at 80 °C for 2 h. The residue was extracted once more under the same conditions. The combined methanol extracts were evaporated to dryness under reduced pressure.

1.3. Liquid-liquid partitioning

The dried residue was dissolved in ultrapure water (200 mL).

Flavonoid fraction: The aqueous solution was extracted three times with ethyl acetate (3 × 150 mL). The combined ethyl acetate layers were evaporated to dryness.

Saponin fraction: The remaining aqueous layer was then extracted three times with water-saturated n-butanol (3 × 150 mL). The combined n-butanol layers were evaporated to dryness.

Polysaccharide fraction: The final aqueous layer was concentrated, and four volumes of anhydrous ethanol were added (final ethanol concentration ~80 %). The mixture was kept at 4 °C for 24 h, then centrifuged (5000 rpm, 10 min). The precipitate was washed with ethanol, redissolved in water, and freeze-dried.

2. Establishment of an HPLC method for flavonoids

The results of non-targeted metabolomics experiments in the early stage of this study showed that the contents of calycosin-7-glucoside, calycosin, ononin, and formononetin changed significantly before and after fermentation. Therefore, in this experiment, the changes in calycosin-7-glucoside, calycosin, ononin, and formononetin in different fermentation processes of Astragali radix and Huaier were quantitatively analyzed.

2.1. Investigation of linear relationship

The standard solution of calycosin-7-glucoside, ononin, calycosin and formononetin were prepared as reference solutions. The injection volume was 10 μL each time, which was injected into the high-performance liquid chromatograph, and the peak area was measured. The linear regression equation

was made with the chromatographic peak area of the standard as the ordinate (y) and the concentration of the reference substance as the abscissa (x). The results are shown in **Table S11**, and R^2 was greater than 0.999, indicating a good linear relationship.

Table S11. Results of linear relationship investigation.

Component	Regression equation*	R^2	range of linearity（mg·mL-1）
calycosin-7-glucoside	y = 3E+7x - 23098	0.9994	0.0005~0.1
Ononin	y = 5E+7x - 32680	0.9995	0.0005~0.1
Calycosin	y = 4E+7x - 25908	0.9997	0.0005~0.1
formononetin	y = 5E+7x - 25736	0.9999	0.0005~0.1

*: x, Standard solution concentration, mg·mL-1; y, Peak area.

2.2. Precision investigation

The standard solution of the same mass concentration was taken and injected continuously for 6 times according to the conditions of ' 2.5.2 ', 10 μL each time. The peak area was recorded, and RSD was calculated. The results are shown in **Table S12.** The RSD is less than 5 %, indicating that the instrument has good precision.

Table S12. Precision investigation results.

Serial number	calycosin-7-glucoside	Ononin	Calycosin	formononetin
1	145109	192638	213446	258291
2	144164	189825	210309	254659
3	145787	191970	213652	257242
4	146065	192637	213395	256921
5	145643	192357	213118	258664
6	146194	192663	213921	258793
Average	145493.66	192015.00	212973.50	257428.33
RSD	0.47%	0.53%	0.57%	0.55%

2.3. Stability study

Test solutions of the fungal substances and sterilized decoction pieces were injected at 0,2,4,8,12 and 24 h, 10 μL each time. The peak area was recorded, and the RSD value was calculated. The results are shown in **Table S13** indicating that the test solution of fungal substances and sterilized decoction pieces had good stability within 24 h.

2.4. Reproducibility study

Six portions of fungal substance and sterilized decoction pieces powder were taken, and six portions of fungal substance and sterilized decoction pieces were prepared. The samples were injected, and the injection volume was 10 μL. The peak area was recorded, and the RSD value was calculated. The results are shown in **Table S14**, indicating good reproducibility.

2.5. Sample recovery investigation

Six portions of fungal substance and sterilized decoction pieces powder were taken, and twice the amount of calycosin-7-glucoside, calycosin, ononin and formononetin reference substance was added accurately. Six portions of fungal substance and sterilized decoction pieces were prepared. The samples and the injection volume were 10 μL. The peak area was recorded, and the RSD value was calculated. The results are shown in **Table S15** and **Table S16**, indicating that the recovery rate was good.

Table S13. Stability test results.

	Mycoplasm				Medical herb in pieces			
Time	calycosin-7-glucoside	Calycosin	Ononin	formononetin	calycosin-7-glucoside	Calycosin	Ononin	formononetin
0 h	1677744	645384	1126102	818292	25800	16314	8623	9869
2 h	1688572	659527	1142603	824426	26356	15977	8676	9688
4 h	1697803	652178	1138294	826530	25973	16920	8863	10246
8 h	1694778	653543	1139680	829684	25802	16547	8818	10829
12 h	1697081	657273	1133513	820764	25671	15668	8876	10347
24 h	1680417	650976	1138757	820180	25417	15659	8889	10172
Average	1689399	653147	1136492	823313	25837	16181	8791	10192
RSD	0.47%	0.70%	0.47%	0.48%	1.11%	2.85%	1.18%	3.56%

Table S14. Reproducible results.

	Mycoplasm				Medical herb in pieces			
Number	calycosin-7-glucoside	Calycosin	Ononin	formononetin	calycosin-7-glucoside	Calycosin	Ononin	formononetin
1	613758	494368	925029	840838	25639	15349	8482	9869
2	635327	491195	913198	826205	25800	16314	8623	9391
3	603910	484483	916836	821774	25973	16920	8863	10246

Number	Mycoplasm calycosin-7-glucoside	Calycosin	Ononin	formononetin	Medical herb in pieces calycosin-7-glucoside	Calycosin	Ononin	formononetin
4	630430	486132	917323	816882	24123	16451	8691	9591
5	628634	498367	930579	833645	26356	15977	8676	9688
6	631608	484246	928133	831336	24520	15368	8989	9584
Average	1684330	650503	1137053	824490	25402	16063	8721	9728
RSD	0.66%	1.16%	0.82%	1.40%	3.16%	3.55%	1.88%	2.79%

Table S15. Investigation into the recovery of bacterioplasm by adding samples.

		calycosin-7-glucoside		Ononin		Calycosin		formononetin	
Number	Sampling volume（g）	Adding quantity（mg）	Recovery rate	Adding quantity（mg）	Recovery rate	Adding quantity（mg）	Recovery rate	Adding quantity（mg）	Recovery rate
1	1	0.569	98.06%	0.108	100.86%	0.291	97.40%	0.170	97.35%
2	1	0.569	101.47%	0.108	96.83%	0.291	104.11%	0.170	103.33%
3	1	0.569	95.68%	0.108	100.79%	0.291	98.83%	0.170	98.96%
4	1	0.569	97.21%	0.108	96.61%	0.291	102.73%	0.170	101.37%
5	1	0.569	102.53%	0.108	103.66%	0.291	103.22%	0.170	101.53%

		calycosin-7-glucoside		Ononin		Calycosin		formononetin	
6	1	0.569	96.31%	0.108	98.06%	0.291	96.90%	0.170	95.95%
Average			98.54%		99.47%		100.53%		99.75%
RSD			2.54%		2.54%		2.89%		2.57%

Table S16. Study on the recovery of decoction pieces by adding samples.

		calycosin-7-glucoside		Ononin		Calycosin		formononetin	
Number	Sampling volume（g）	Adding quantity（mg）	Recovery rate	Adding quantity（mg）	Recovery rate	Adding quantity（mg）	Recovery rate	Adding quantity（mg）	Recovery rate
1	1	0.016	102.90%	0.007	104.75%	0.009	100.21%	0.007	100.65%
2	1	0.016	96.19%	0.007	96.64%	0.009	99.61%	0.007	103.98%
3	1	0.016	97.44%	0.007	95.46%	0.009	95.39%	0.007	100.28%
4	1	0.016	96.07%	0.007	100.66%	0.009	95.70%	0.007	104.36%
5	1	0.016	98.54%	0.007	97.51%	0.009	95.80%	0.007	101.06%
6	1	0.016	98.73%	0.007	104.64%	0.009	100.43%	0.007	99.51%
Average			98.31%		99.94%		97.86%		101.64%
RSD			2.33%		3.71%		2.29%		1.82%

3. Establishment of HPLC method for saponin components

The results of non-targeted metabolomics showed that the contents of astragaloside I, astragaloside II, astragaloside III and astragaloside IV changed significantly, and cycloastragenol glucoside was produced with the fermentation. Therefore, the experiment chooses to quantitatively analyze the changes in astragaloside I, astragaloside II, astragaloside III, astragaloside IV, cycloastragenol-6-glucoside, and cycloastragenol in the fermentation process of astragali radix and huaier.

3.1. Investigation of linear relationship

Astragaloside I, astragaloside II, astragaloside III, astragaloside IV, cycloastragenol glucoside, and cycloastragenol reference substances were injected into the high-performance liquid chromatograph, respectively. The injection volume was 10 μL each time, and the peak area was determined. Because the peak area of the evaporative light detector was not proportional to the concentration, the logarithm was taken to investigate the linear relationship. In this experiment, the logarithm of the chromatographic peak area of the standard was used as the ordinate (y), and the logarithm of the concentration of the reference substance was used as the abscissa (x) to do the linear regression equations. The results are shown in **Table S17**. R^2 was greater than 0.999, indicating a good linear relationship.

Table S17. Results of linear relationship investigation.

Component	Regression equation*	R^2	Range of linearity（$mg\cdot mL^{-1}$）
Astragalus saponin I	y = 1.4848x + 6.1784	0.9992	0.0031-0.1
Astragalus saponin II	y = 1.5205x + 6.3106	0.9991	0.0031-0.1
Astragalus saponin III	y = 1.2614x + 5.8071	0.9991	0.0031-0.1
Astragaloside	y = 1.4784x + 6.2961	0.9998	0.0031-0.1
Cycloastragenol glucoside	y = 1.4961x + 6.252	0.9991	0.0031-0.1

*: x, Logarithm of the standard solution concentration, mg/mL; y, Logarithm of the peak area.

3.2. Investigation of precision

The standard solution of the same mass concentration was taken and repeated 6 times, 10 μL each time. The peak area was recorded, and the precision was calculated. The results were as follows: **Table S18**, RSD was less than 5 %, indicating that the precision of the instrument was good.

Table S18. Precision investigation results.

Number	Astragaloside	Astragalus saponin II I	Astragalus saponinII	Cycloastragenol glucoside	Astragalus saponinI
1	21629	14639	21282	18433	16898
2	21908	14631	22252	19002	16470
3	22527	14676	21890	18920	16768
4	21836	13757	22793	18453	15972
5	22702	14629	23083	18891	16951
6	22003	13828	22326	19015	15344
Average	22100.833	14360.000	22271.000	18785.667	16400.500
RSD	1.74%	2.80%	2.63%	1.31%	3.51%

3.3. Stability investigation

The test solution of fungal substance and sterilized decoction pieces was injected at 0,2,4,8,12 and 24 h according to the conditions, 10 μL each time. The peak area was recorded, and the RSD value was calculated. The results are shown in **Table S19**, indicating that the test solution of fungal substance and sterilized decoction pieces had good stability within 24 h.

3.4. Reproducibility study

Six samples of fungal substance and six samples of sterile decoction pieces were prepared, and six samples of fungal substance and six samples of sterile decoction pieces were prepared. The injection volume was 10 μL, the peak area was recorded, and the RSD value was calculated. The results showed that the reproducibility was good, as shown in **Table S20**.

3.5. Sample recovery investigation

Six portions of fungal substance and sterilized decoction pieces powder were taken, and twice the amount of astragaloside I, astragaloside II, astragaloside III, astragaloside IV, and cycloastragaloside reference substance was added accurately. Six portions of fungal substance and sterilized decoction pieces were prepared. The samples were injected according to the conditions, and the injection volume was 10 μL. The peak area was recorded, and the RSD value was calculated. The results are shown in **Table S21** and **Table S22**, indicating that the recovery rate was good.

Table S19. Stability test results.

	Fungal substance		Astragali radix			
Time	Cycloastrageno l glucoside	Astragalus saponinI	Astragaloside	Astragalus saponin II I	Astragalus saponinII	Astragalus saponinI
0 h	16912	724	1177	1195	5436	17121
2 h	16750	723	1246	1164	5399	16908
4 h	16952	715	1206	1250	5340	17232
8 h	17597	730	1224	1185	5425	15948
12 h	16099	720	1173	1131	5490	16690
24 h	17161	701	1191	1182	5351	17002
Average	16911.833	718.833	1202.833	1184.500	5406.833	16816.833
RSD	2.67%	1.27%	2.15%	3.02%	0.95%	2.52%

Table S20. Reproducible results.

	Fungal substance		Astragali radix			
Number	Cycloastragenol glucoside	Astragalus saponinI	Astragaloside	Astragalus saponinⅡI	Astragalus saponinII	Astragalus saponinI
1	15524	736	1221	1232	5550	17284
2	16912	709	1219	1273	5413	17155
3	16952	719	1206	1250	5340	17232
4	16084	724	1257	1171	5370	17897
5	15854	723	1246	1164	5399	16908
6	16750	715	1177	1195	5436	17121
Average	16346.000	721.000	1221.000	1214.167	5418.000	17266.167
RSD	3.39%	1.16%	2.14%	3.33%	1.23%	1.77%

Table S21. Investigation into the recovery of bacterioplasm by adding samples.

		Cycloastragenol-6-O-glucoside		Astragaloside I	
Number	Sampling quantity（g）	Adding quantity（mg）	Recovery rate	Adding quantity（mg）	Recovery rate
1	1	0.434	104.13%	0.058	101.67%
2	1	0.434	102.42%	0.058	102.73%
3	1	0.434	102.16%	0.058	103.59%
4	1	0.434	104.58%	0.058	101.53%
5	1	0.434	100.52%	0.058	100.40%
6	1	0.434	103.18%	0.058	103.33%
Average			102.83%		102.21%
RSD			1.31%		1.09%

Table S22. Study on the recovery of decoction pieces by adding samples.

		Astragaloside		Astragalus saponin ⅡI		Astragalus saponinII		Astragalus saponinI	
Number	sample size（g）	Adding quantity（mg）	Recovery rate	Adding quantity（mg）	Recovery rate	Adding quantity（mg）	Recovery rate	Adding quantity（mg）	Recovery rate
1	1	0.067	101.24%	0.069	100.23%	0.202	96.65%	0.493	96.07%
2	1	0.067	95.80%	0.069	97.43%	0.202	102.17%	0.493	102.56%
3	1	0.067	100.40%	0.069	96.16%	0.202	98.09%	0.493	103.78%
4	1	0.067	99.44%	0.069	98.58%	0.202	97.55%	0.493	95.05%
5	1	0.067	104.23%	0.069	102.25%	0.202	100.87%	0.493	96.20%
6	1	0.067	100.44%	0.069	97.81%	0.202	97.86%	0.493	98.31%
Average			100.26%		98.74%		98.86%		98.66%
RSD			2.49%		2.02%		1.99%		3.40%

S3. Establishment of a Method for Determining Polysaccharide Content

1. Examination of Linear Relationships

A linear regression equation was established with the absorbance values of the glucose reference solution plotted on the y-axis and the reference concentration on the x-axis, as shown in **Figure S6.** The linear equation is $y = 7.9786x - 0.017$, with $R^2 = 0.999$, indicating a good linear relationship. The linear range is 0.01–0.1 mg·mL^{-1}.

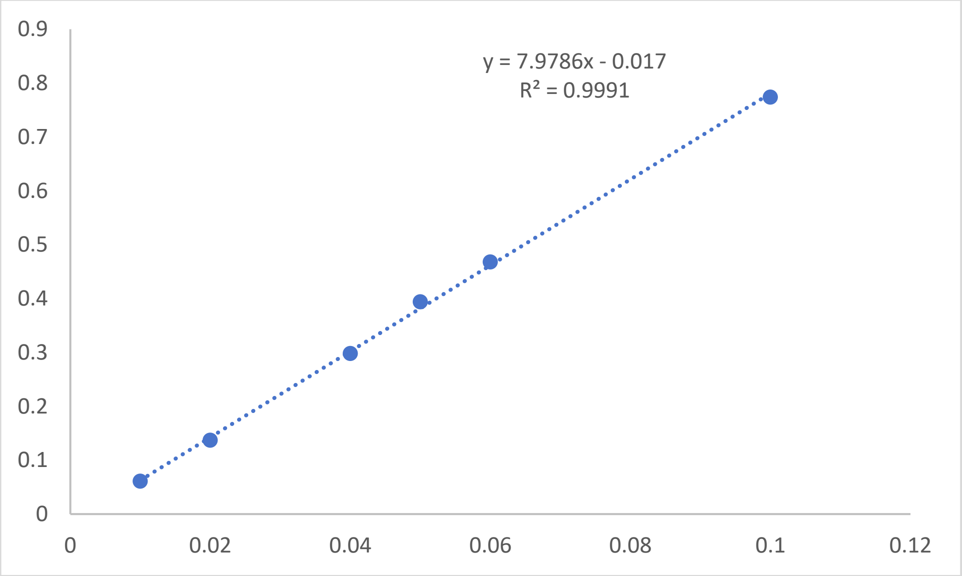

Figure S6. Results of the linear relationship study for the glucose standard.

2. Precision Examination

Using a standard solution of the same mass concentration, six consecutive determinations were performed under the conditions specified in Section 1. The relative standard deviation (RSD) values were calculated, with results shown in **Table S23**. An RSD of less than 5% indicates good precision.

Table S23. Precision investigation results.

Number of times	1	2	3	4	5	6	Average	RSD
Absorbance	0.468	0.470	0.469	0.469	0.470	0.471	0.470	0.22%

3. Stability Assessment

Test solutions were prepared from the fungal material and sterilized herbal slices. Determinations were conducted at 0, 15, 30, 45, and 60 minutes under the conditions specified in Section 1. The relative standard deviation (RSD) values were calculated, with results presented in **Table S24**. An RSD below 5% indicates satisfactory stability of the samples within one hour.

Table S24. Stability test results.

Time（min）		0	15	30	45	60	Average	RSD
Absorbance	Herbal powder	1.22	1.154	1.134	1.163	1.147	1.164	2.90%
	Fungal material	0.653	0.659	0.655	0.675	0.649	0.658	1.50%

4. Reproducibility assessment

Take 6 g each of fungal material and sterilized herbal powder. Determine according to the conditions specified under "1.", calculate the RSD value, and the results are shown in **Table S25**, indicating good reproducibility.

Table S25. Reproducibility test results.

Sample		1	2	3	4	5	6	Average	RSD
Absorbance	Herbal powder	1.218	1.136	1.147	1.162	1.111	1.194	1.161	3.40%
	Fungal material	0.593	0.633	0.653	0.64	0.594	0.616	0.622	4.00%

5. Sample Recovery Assessment

Take six portions each of fungal material and sterilized herbal powder, precisely add an equal volume of glucose, and determine according to the conditions under "1.". Calculate the RSD value; the results are shown in **Table S26**, indicating satisfactory recovery rates.

Table S26. Sample recovery and determination results.

		Fungal material		Herbal powder	
NO	Weighing capacity（g）	Glucose addition amount（mg）	Sample recovery rate	Glucose addition amount（mg）	Sample recovery rate
1	1	80	99.09%	148	104.14%
2	1	80	98.00%	148	101.00%
3	1	80	104.58%	148	96.85%
4	1	80	102.54%	148	102.11%
5	1	80	100.19%	148	100.24%
6	1	80	97.53%	148	99.06%
Average			100.32%		100.57%
RSD			2.74%		2.49%

Table S27. Binding free energies and energy components predicted by MM/GBSA (kcal/mol).

System name	XOD /Cycloastragenol-6-O-β-D-glucoside
ΔE_{vdw}	-45.30±3.70
ΔE_{elec}	-43.40±7.70
ΔG_{GB}	66.47±6.86
ΔG_{SA}	-6.42±0.43
ΔG_{bind}	-28.66±4.04

(Note: ΔEvdW: van der Waals energy; ΔEelec: electrostatic energy; ΔGGB: electrostatic contribution to solvation; ΔGSA: non-polar contribution to solvation; ΔGbind: binding free energy.)

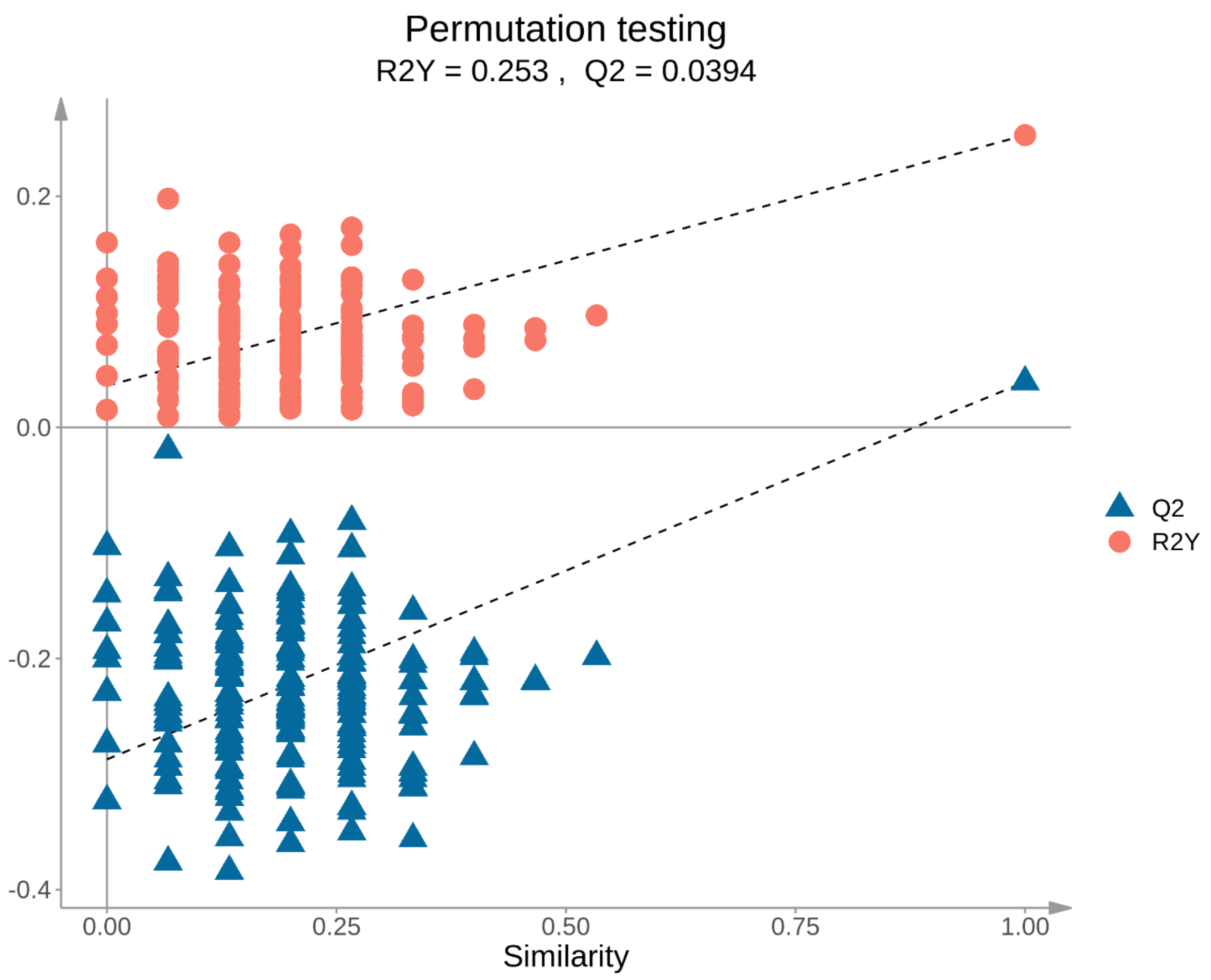

Figure S7. Permutation plot of the OPLS-DA diagram.

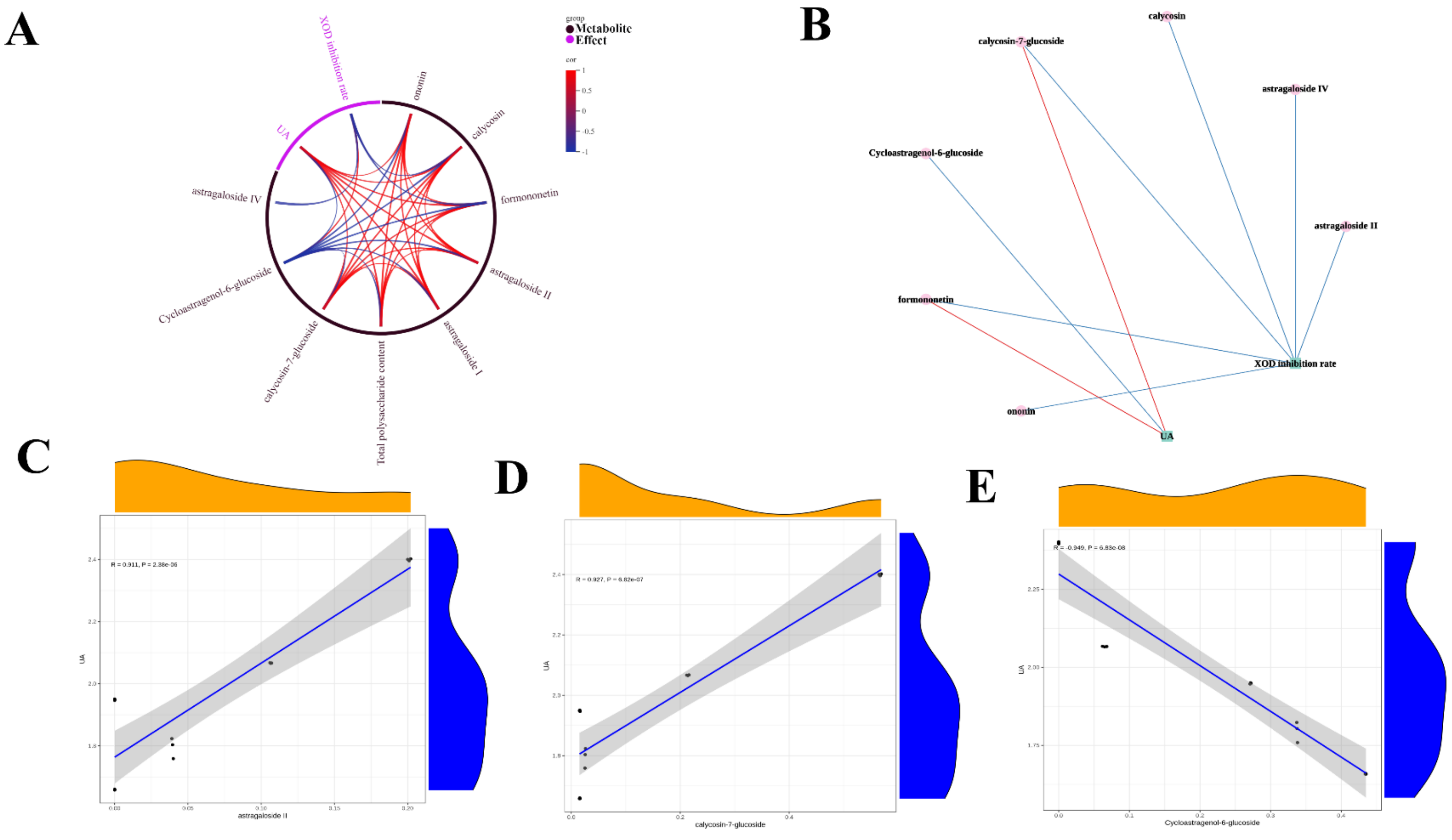

Figure S8. Metabolism-pharmacodynamic correlation analysis. (A-B): Metabolism-pharmacodynamic correlation ring diagram and network. (C-E) Pearson correlation and linear regression for Cycloastragenol-6-glucoside, calycosin-7-glucoside, astragaloside II vs. UA.

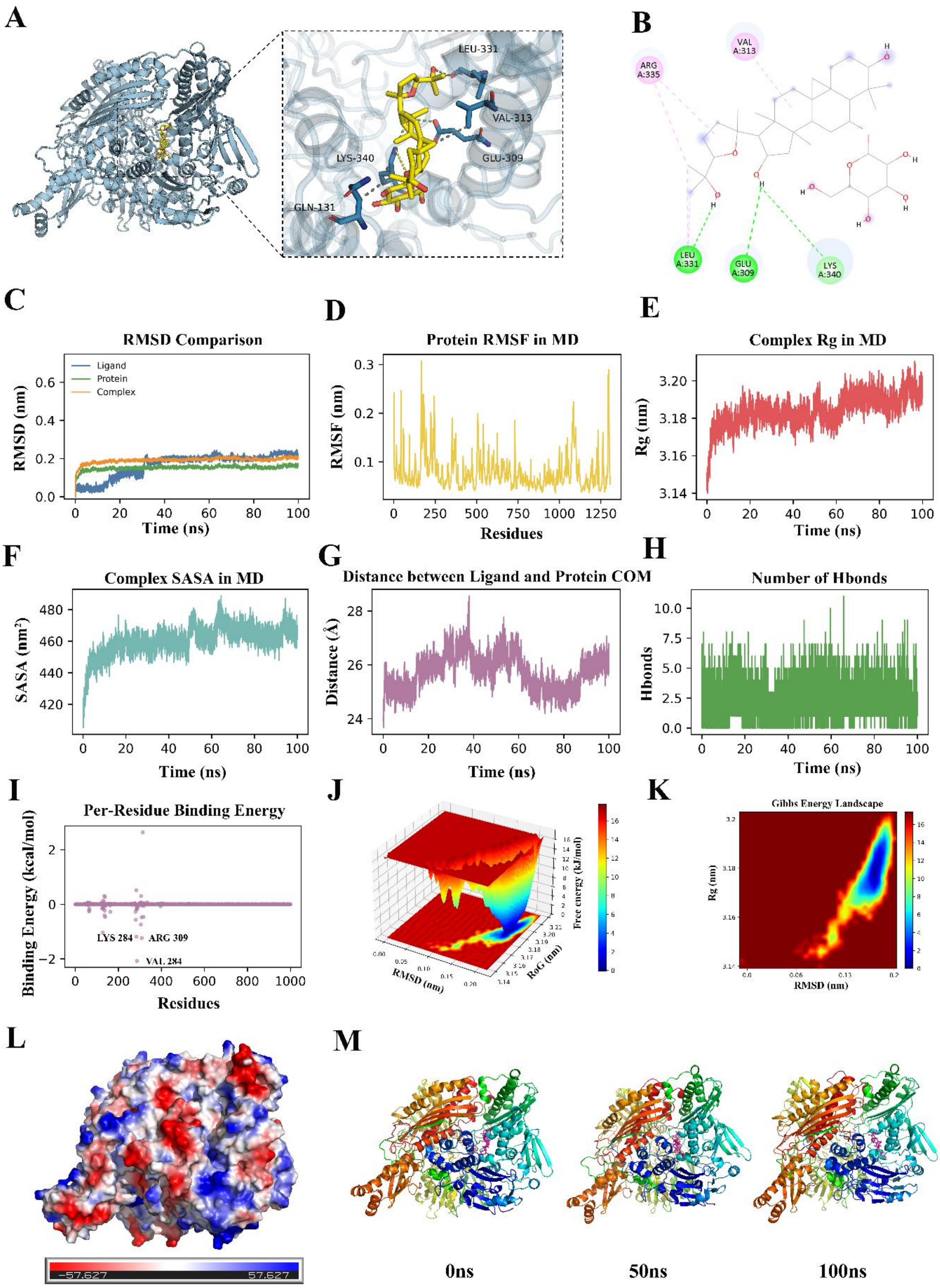

Figure S9. Molecular dynamics analysis of the interaction between Cycloastragenol-6-O-β-D-glucoside and XOD.

(A): Overall binding conformation of the small molecule in the protein binding pocket. (B): Schematic diagram of the two - dimensional interaction between the ligand and key amino acid residues. (C): RMSD changes of the ligand, receptor, and complex during the 100 ns

molecular dynamics simulation. (D): RMSF distribution of the protein backbone residues. (E): Changes in the radius of gyration (Rg) of the complex during the simulation. (F): Changes in the solvent - accessible surface area (SASA) of the complex over time. (G): Changes in the distance between the centroid of the ligand and the centroid of the protein. (H): Changes in the number of hydrogen bonds between the protein and the ligand during the simulation. (I): Energy contribution analysis of key amino acid residues to the binding free energy. (J): Three - dimensional free energy landscape (FEL) constructed based on RMSD and Rg. (K): Two - dimensional projection map of the free energy landscape. (L): Electrostatic potential distribution map on the protein surface. (M): Conformational change diagrams of the complex at 0 ns, 50 ns, and 100 ns during the molecular dynamics simulation.
